# Regulation of Lipid Dysmetabolism and Neuroinflammation Progression Linked With Alzheimer's Disease Through Modulation of Dgat2

**DOI:** 10.1111/acel.70439

**Published:** 2026-03-15

**Authors:** Archana Yadav, Xiaosen Ouyang, Morgan Barkley, John C. Watson, Kishore Madamanchi, Josh Kramer, Jianhua Zhang, Girish C. Melkani

**Affiliations:** ^1^ Department of Pathology, Division of Molecular and Cellular Pathology, Heersink School of Medicine University of Alabama at Birmingham Birmingham Alabama USA; ^2^ UAB Nathan Shock Center Birmingham Alabama USA

**Keywords:** aging, Alzheimer's disease, amyloid‐β42, diacylglycerol O‐acyltransferase 2, *Drosophila *and mouse models, lipid metabolism, sleep‐circadian rhythms, neuroinflammation

## Abstract

Alzheimer's disease (AD), an age‐associated neurodegenerative disorder, is characterized by progressive cognitive decline, amyloid‐β (Aβ) accumulation (including soluble oligomers and deposited aggregates), lipid dysregulation, and neuroinflammation. Although mutations in the amyloid precursor protein (APP) and accumulation of Aβ42 are established drivers of pathology, the mechanisms connecting oligomeric amyloid toxicity with lipid metabolism and inflammatory responses remain poorly understood. Here, we employed complementary *Drosophila* and mouse models to dissect these relationships. Panneuronal, glial or mushroom body specific expression of humanized *App* ^*NLG*^ and Aβ42 in *Drosophila* resulted in locomotor deficits, disrupted sleep–circadian rhythms, memory impairments, lipid accumulation, synaptic loss, and neuroinflammatory signatures. Comparable lipid accumulation, metabolic dysregulation and neuroinflammation were detected in the *App* ^*NLG‐F*^ knock‐in mouse model, underscoring their conserved relevance to AD pathogenesis. We further identified diacylglycerol O‐acyltransferase 2 (Dgat2), a key enzyme catalyzing the final step of triglyceride synthesis, as a critical modulator of AD‐related phenotypes. *Dgat2* expression was altered in both animal models and human AD tissues. Notably, panneuronal knockdown of *Dgat2* in *Drosophila* attenuated lipid accumulation, restored synaptic integrity, and ameliorated locomotor and cognitive deficits, while also reducing neuroinflammation. Additionally *Dgat2* suppression improved sleep and circadian behavior, highlighting its pleiotropic protective effects. Together, these findings support a mechanistic link between amyloid pathology, lipid dysregulation, and neuroinflammatory processes. The conservation of lipid homeostasis mechanisms across species underscores the translational potential of this approach for delaying or mitigating AD progression. Moreover, targeting Dgat2 may therefore represent a novel therapeutic strategy to counteract AD‐associated metabolic and neuronal dysfunction.

## Graphical abstract

*Drosophila* and mouse models of Alzheimer's disease exhibit lipid accumulation and neuroinflammation, leading to progressive impairments in memory, locomotion, sleep, circadian rhythms, and synaptic integrity. *Knockdown* of *Dgat2* reduces lipid accumulation and neuroinflammatory signaling, rescuing these deficits and highlighting a conserved, crossspecies therapeutic strategy targeting lipid metabolism in age‐related Alzheimer's disease.

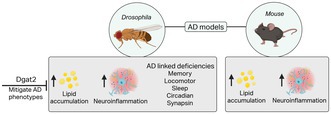

**Abbreviations**
ABCA1: ATP‐binding cassette transporter A1
ACC: acetyl‐CoA carboxylase
AD: Alzheimer's disease
ApoE: apolipoprotein E
APP: amyloid precursor protein
Aβ: amyloid‐β
BMP: bis‐phosphates
*Dgat2*
: *diacylglycerol O‐acyltransferase 2*
*Dgat2 KD*
: *diacylglycerol O‐acyltransferase 2* *knockdown*
FAS: fatty acid synthase
GWAS: genome‐wide association studies
PSEN1: presenilin1
PSEN2: presenilin2
Srebp: sterol regulatory element binding protein
TREM2: triggering receptor expressed on myeloid cells 2
TRF: time‐restricted feeding
WT: wild‐type

## 1 Introduction

Alzheimer's disease (AD) is a progressive neurodegenerative disorder that primarily affects older adults and is the leading cause of dementia worldwide (Collaborators, G. B. D. D. F 2022; Western et al. 2024). While the exact causes of AD are not fully understood, genetic mutations play a significant role, especially in early‐onset familial forms of the disease. Three rare single‐gene variants, amyloid precursor protein (APP), presenilin 1 (PSEN1), and presenilin 2 (PSEN2), are responsible for these familial cases. APP encodes the amyloid‐β precursor protein, which, when cleaved by enzymes such as β‐secretase and γ‐secretase, generates amyloid‐β (Aβ). These Aβ peptides assemble into soluble oligomers and higher‐order aggregates (including plaques), pathological features found in AD brains. Extensive evidence indicates that soluble Aβ oligomer drive synaptic dysfunction as principal drivers of early functional impairment, whereas plaque burden shows a limited correlation with cognitive decline. Consistently, soluble Aβ oligomers rather than insoluble plaques are strongly linked to synaptic loss and cognitive decline (Selkoe 2008; Shankar et al. 2008). Despite decades of research, the precise molecular mechanisms driving the AD pathogenesis remain unclear, and there is still no effective and safe treatment to slow or halt its progression (Bartoletti‐Stella et al. 2018; Uddin et al. 2020). In addition to oligomeric Aβ accumulation, neuroinflammation plays a critical role in the development of AD, with elevated pro‐inflammatory cytokines, reduced anti‐inflammatory responses, and heightened glial reactivity (Bellaver et al. 2023). Lipids, particularly fatty acids and their derivatives, have long been recognized for their immunomodulatory effects, capable of triggering either pro‐inflammatory or anti‐inflammatory responses depending on the lipid class and the target cell (Daynes and Jones 2002; Leng and Edison 2021). Additionally, there is growing evidence of altered lipid profiles and lipid accumulation associated with Aβ pathology in human AD brains. Lipid dysregulation may significantly influence the development and toxicity of soluble Aβ oligomers, as the composition of membrane lipids impacts oligomer assembly, stability, and interaction with synaptic membranes (Bode et al. 2019; He et al. 2025; Mirdha 2024).

As one of the critical aspects of AD pathogenesis, altered lipid profiles and lipid accumulation have been found in both animal models and postmortem human AD brains (Munkacsy et al. 2019; Ralhan et al. 2021). Several lipid‐related genes have been identified as genetic risk factors for AD, including apolipoprotein E (ApoE), ATP‐binding cassette transporter A1 (ABCA1), phosphatidylinositol‐binding clathrin assembly protein (PICALM), and triggering receptor expressed on myeloid cells 2 (TREM2) (Martens et al. 2022; Picard et al. 2018). Notably, the ApoE ɛ4 allele is a well‐established genetic risk factor for late‐onset AD, with ApoE playing a crucial role in lipid transport and neuronal integrity (Chen et al. 2024). Specifically, a *neurolipid atlas* demonstrates that ApoE ɛ4‐driven cholesterol ester accumulation in astrocytes is accompanied by impaired immune signaling, reinforcing the link between lipid metabolism and neuroinflammation in AD (Feringa et al. 2025). The upregulation of enzymes like fatty acid synthase (FAS) and acetyl‐CoA carboxylase (ACC) in AD models suggests disrupted lipid synthesis, with palmitic acid (C16) accumulation in regions of Aβ pathology, highlighting lipid pathways as potential therapeutic targets in AD (Ates et al. 2020; Daugherty et al. 2017). In addition to neurons, astrocytes primarily degrade fatty acids in the brain. Their metabolic role in AD and neurodegeneration is increasingly recognized with recent studies suggesting that impaired astrocytic fatty acid degradation may contribute to lipid dysregulation in the brain (Daugherty et al. 2017). Both microglia and astrocytes express enzymes for β‐oxidation, with fatty acid metabolism influencing microglial phenotype and inflammation. Inhibition of β‐oxidation in macrophages and microglia exacerbates inflammation, while enhancing fatty acid oxidation reduces lipid‐induced inflammation. Activation of mitochondrial function in microglia helps alleviate neuroinflammation and promotes amyloid‐β clearance (Liu et al. 2019; Namgaladze and Brune 2016; Song and Suk 2017). In transgenic mice expressing mutant APP, there is an increase in Aβ42 oligomers and aggregates in the brain along with elevated plasma cholesterol levels, further supporting the involvement of disrupted lipid metabolism in AD pathogenesis (Barbero‐Camps et al. 2013). Elevated levels of lysophospholipids and ceramides have been detected in regions of Aβ deposition in the human AD brain. Similarly, in *App* ^*NLG‐F*^ knock‐in mice, an age‐dependent increase in lysophospholipids and bisphosphates has been observed in association with Aβ pathology (Huang et al. 2024). Importantly, membrane lipid composition, especially cholesterol, sphingomyelin, and gangliosides, affects the production of oligomers and their insertion into membranes, potentially contributing to synaptic toxicity (Toprakcioglu et al. 2025; Zhang et al. 2022). Despite these findings, the precise role of lipid metabolism in oligomer mediated toxicity and AD pathology remains underexplored. Understanding how lipid dysregulation contributes to oligomeric Aβ toxicity could open new avenues for therapeutic intervention. Recent advances, such as the development of a *neurolipid atlas* that integrates cell‐type–specific lipidomic profiles with neurodegenerative disease pathways, provided critical insights into how lipid dysregulation intersects with inflammatory signaling in AD (Feringa et al. 2025). This resource underscores the complexity of lipid metabolism in AD and supports the need for mechanistic studies, to clarify how oligomeric Aβ toxicity and lipid pathways converge.

One of the key enzymes involved in lipid metabolism is diacylglycerol O‐acyltransferase 2 (Dgat2), which catalyzes the final step in triglyceride synthesis (Adamovich et al. 2014). Interestingly, *Dgat2* has been implicated in both lipid metabolism and the regulation of circadian rhythms, suggesting that its role extends beyond simple lipid storage and may influence the timing of metabolic processes (Livelo et al. 2023, 2025; Moraes et al. 2024). *Dgat2* levels are increased in human AD brains and mouse AD models (Prakash et al. 2023), with single‐nucleus RNA sequencing (snRNA‐seq) demonstrating increased expression in excitatory neurons (Brase et al. 2023). Inhibition of DGAT2 activity in microglia has been reported to improved microglial uptake of Aβ (Prakash et al. 2025). In 5xFAD mice, a one‐week treatment with proteasome activator reported to reduce DGAT2 levels was associated with decreased APP immunoreactivity in the subiculum (Prakash et al. 2025). However, whether the proteasome activator selectively degrades microglia Dgat2 remains unclear, and it is also unknown whether memory function is improved by DGAT2 degradation. Despite these observations, the precise meachanism by which Dgat2 contributes to AD‐associated lipid dysregulation and neuroinflammation remain unclear.

Elevated levels of lysophospholipids and ceramides near amyloid plaques have been observed in AD, consistent with lipid remodeling in plaque‐associated pathology (Huang et al. 2024). AD is also linked with significant alterations in membrane phospholipid composition, which can affect lipid bilayer structure, oligomer assembly and inflammatory signaling, in addition to lipid droplets formation. The AD brain shows an intense loss of phosphatidylethanolamine (PtdEtn) plasmalogens, which serve as endogenous antioxidants and help preserve membrane fluidity (Dorninger et al. 2020; Wood et al. 2015). Furthermore, disruption of lipid bilayer asymmetry occurs, leading to the externalization of phosphatidylserine (PS) from the inner to the outer membrane leaflet, a change typically associated with apoptotic signaling (Gomes et al. 2022; Mapstone et al. 2014). These alterations in membrane composition have direct inflammatory consequences; depletion of PtdEtn plasmalogens heightens vulnerability to oxidative stress and lipid peroxidation, resulting in the formation of reactive lipid species that activate glial inflammatory pathways (Chew et al. 2020; Dorninger et al. 2020). Moreover, PS externalization functions as an “eat‐me” signal, initiating microglial activation and phagocytic responses, while simultaneously promoting reactive astrocyte production via lipid‐mediated signaling cascades (Joshi et al. 2019; Liddelow et al. 2017). Recent lipidomic studies, including extensive resources like the *neurolipid atlas*, have shown that these phospholipid modifications reveal early in AD pathogenesis and correlate with inflammatory markers across multiple neurodegenerative conditions (Bennett et al. 2013; Emre et al. 2021; Feringa et al. 2025). Reduction in PtdEtn plasmalogens and altered phospholipid distribution have been reported in AD brain and may promote cellular stress response and glial activation (Emre et al. 2021; Kao et al. 2020). Taken together, these findings suggest a strong mechanistic connection between lipid metabolism and AD pathology (Moraes et al. 2024).

Circadian rhythms regulate various physiological processes, including sleep, cognition, and metabolism. In AD, circadian rhythms are frequently disrupted, leading to sleep disturbances and cognitive decline. Circadian rhythms influence lipid metabolism, and disruptions in circadian timing can lead to metabolic dysfunction, including altered lipid storage and degradation (Austad et al. 2022; Hardin and Panda 2013; Panda et al. 2002). Furthermore, our previous work in *Drosophila melanogaster* (commonly known as the fruit fly) has implicated lipid metabolism in aging and neurodegeneration (Moraes et al. 2024; Villanueva et al. 2019). We also observed that ApoE induces lipid accumulation in the brain, with ApoE4 uniquely causing this accumulation independently of Dgat2, while ApoE2 and ApoE3 require Dgat2 for lipid buildup (Moraes et al. 2024). These findings highlight the importance of lipid metabolism in AD and suggest that targeting lipid metabolism pathways could mitigate ApoE‐related metabolic dysfunction in AD.

Although growing evidence suggests that lipid dysregulation contributes to the pathogenesis of AD. The molecular role of *Dgat2* mediated triglyceride synthesis in the context of oligomeric Aβ toxicity, neuroinflammation, and synaptic dysfunction remains poorly understood. To address this gap, we utilized *Drosophila* and mouse models to systematically examine how modulation of *Dgat2* affects AD‐related phenotypes across the species. Using panneuronal and cell‐type‐specific expression of *App* ^*NLG*^ and Aβ42 in *Drosophila*, and *App* ^*NLG‐F*^ knock‐in mice that exhibit age‐dependent accumulation of oligomeric Aβ and neurodegeneration, we show the manipulation of *Dgat2* is associated with changes in markedly lipid metabolism, inflammatory processes, synaptic integrity, and behavioral performance. These cross‐species findings establish Dgat2 as a critical node linking lipid dysregulation to oligomer‐mediated toxicity and suggest that targeting lipid metabolic pathways may offer therapeutic potential for mitigating AD progression.

## 2 Results

**FIGURE 1:**
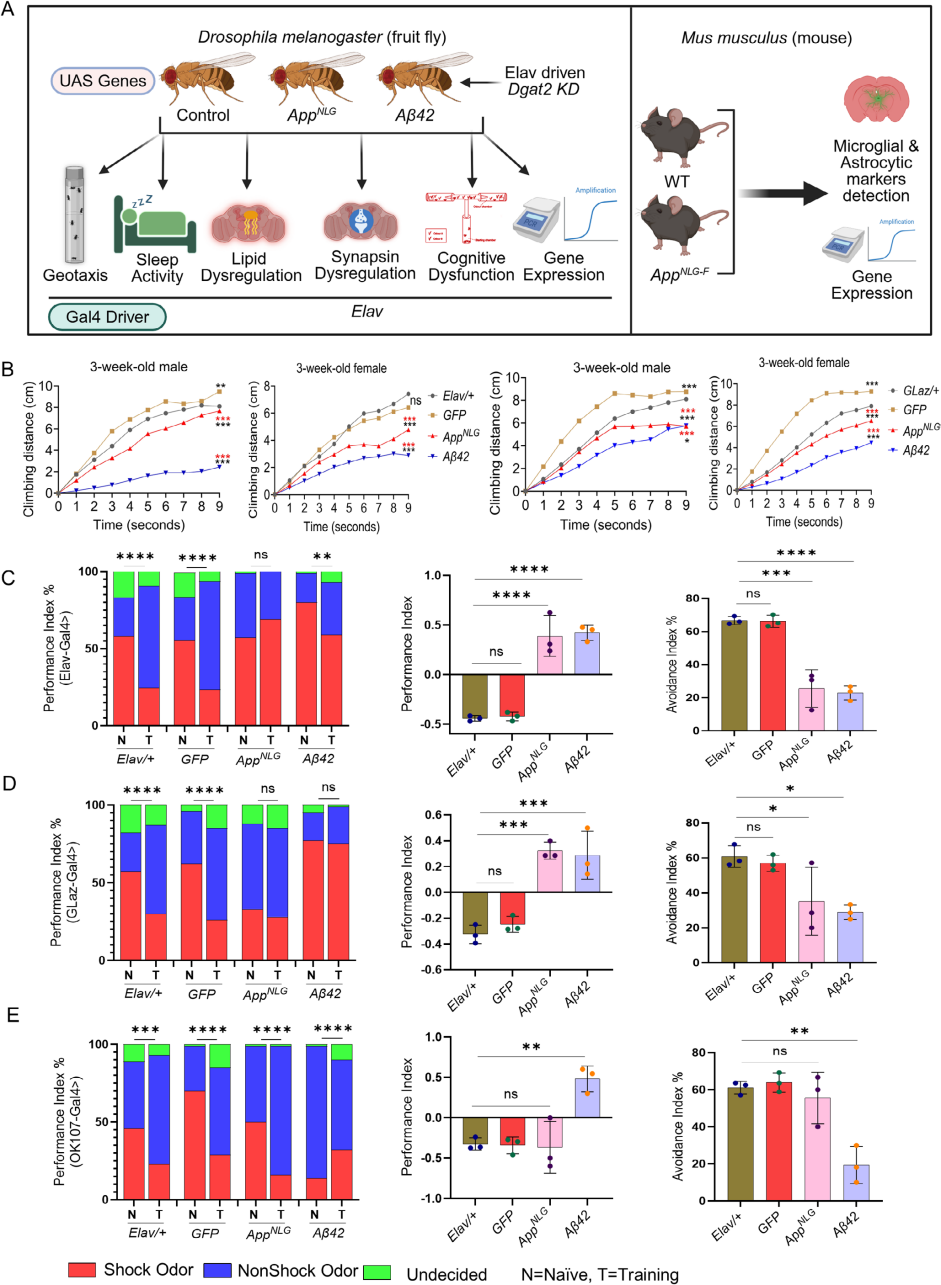
Panneuronal and glial‐specific expression of *App* ^*NLG*^ *and* Aβ42 led to compromised locomotor and cognitive performance. (A) Flow chart depicting the experimental setup. Each UAS gene was crossed with the region‐specific Gal‐4 drivers. To explore the contribution of *Dgat2* in Aβ42 and App accumulation, we performed *Elav*‐driven *Dgat2* *KD* in *Drosophila* and used a young/old *App* ^*NLG‐F*^ mouse model. (B) Negative geotaxis assay of *Elav* and *GLaz* drove A*pp* ^*NLG*^ *and* Aβ42 models in 3‐week‐old males and females (red asterisk denotes significance with GFP, while black asterisks, ns, denotes significance with *Elav/+* and/or *GLaz/+*). (C–E, left panels only) *App* ^*NLG*^ *and* Aβ42 models show deficits in olfactory aversion training in 3‐week‐old male flies in *Elav* (C) *GLaz* (D), and OK107 (E), driven models; data combined from triplicate assays. The significance was based on the chi‐square test. Values are shown as the percentage of flies in each chamber at the end of the assay. (C–E, middle panels only) The difference between fly decisions, in 3‐week‐old male flies in *Elav* (C), *GLaz* (D), and OK107 (E). (C–E, right panels only), flies avoiding shock odor in Elav (C) *GLaz* (D), and OK107 (E), driven *App* ^*NLG*^ *and* Aβ42 models. Data = mean ± SD. One‐way ANOVA with Tukey's multiple comparisons test was performed (Left and right panels only). Two‐way ANOVA with Tukey's multiple comparisons test was performed for geotaxis, *n* = 3 replicates per group, and each group had 10 flies. *p < 0.05, **p < 0.01, ***p < 0.001, and ****p < 0.0001; n.s., not significant (an asterisk denotes significance for the average of all three replicates). Raw data and *p* values are provided in the source data.

A brief outline of our study is shown in Figure 1A to reveal the involvement of amyloid‐β in AD progression using *Drosophila* and mouse models. As described in the method section, we used *Drosophila* models, to examine the effect of UAS*‐App* ^*NLG*^ *and* UAS‐Aβ42 expression in panneuronal (*Elav*), glial (*GLaz*), and mushroom body (OK‐107) drivers. The outline also demonstrated the use of *Dgat2* *knockdown* (*KD*) with these drivers. Negative geotaxis assays were used to assess locomotor performance, and sleep‐circadian activity was conducted using the Drosophila activity monitor (DAM) system to investigate the impact of APP mutation and *Dgat2* modulation. Olfactory aversion training was performed to test memory upon the expression of APP mutants and *Dgat2* modulations. Gene expression analyses of lipid metabolism, neuro‐inflammation, and AD risk genes were conducted. All experiments included appropriate controls (*w* ^1118^ and GFP) at two different ages (Figure 1A). Furthermore, using the *App* ^*NLG‐F*^ mouse model of AD, along with wild‐type control mice, we examined lipid accumulation and glial activation by immunofluorescence. Additionally, quantitative PCR (qPCR) was performed to assess the expression of genes involved in lipid metabolism, neuroinflammation, and AD risk parhways (Figure 1A).

### 2.1 Panneuronal, Glial, or Mushroom‐Body Specific Expression of *App* ^*NLG*^ and Aβ42 Led to Compromised Locomotor and/or Cognitive Performance

As shown in Figure 1B, significant differences in compromised locomotor performance were observed upon expression of *App* ^*NLG*^ and Aβ42, with more pronounced impairments in Aβ42‐expressing flies and more modest effects in *App* ^*NLG*^ females, compared to age‐matched controls in 3‐week‐old flies. Similar trends were seen in male flies with *Elav*‐Aβ42, whereas male flies expressing *the App* ^*NLG*^ construct showed no significant changes in the climbing ability regardless of the driver used. These results suggest that the geotaxis phenotype is driver‐specific and impacts locomotor performance more prominently in females than males, whether through panneuronal or glial expression (Figure 1B). To investigate cognitive performance, we subjected wild‐type, *App* ^*NLG*^, and Aβ42 flies to olfactory aversive conditioning, which tests their ability to associate odors with reinforcers (Figure 1C–E). 3‐week‐old female flies expressing *App* ^*NLG*^ and Aβ42 driven by *Elav* and *GLaz* resulted in significantly impaired cognitive performance (Figure 1C,D left panels). Expression of these AD‐linked genes in the mushroom body using the OK‐107 driver led to even greater deficits in olfactory aversion training (Figure 1E, left panel).

Additionally, we performed two complementary olfactory aversion analyses, measuring odor avoidance (odor avoidance index) and shock acuity (electric shock avoidance index) (Figure 1D,E, middle and right panels, respectively). Both approaches revealed similar conclusions. These findings indicate that the expression of AD‐linked genes in mushroom bodies has a more profound impact on memory compared to panneuronal and glial expression, highlighting the critical role of these brain regions in olfactory learning. This suggests that different brain regions contribute to cognitive dysfunction in AD models, offering new insights into the mechanisms underlying AD‐related memory impairment.

### 2.2 Compromised Sleep‐Circadian Activity in Flies With Panneuronal and Glial‐Specific Expression of Aβ42 and in Aged Glial‐Specific Expression of *App* ^*NLG*^

**FIGURE 2:**
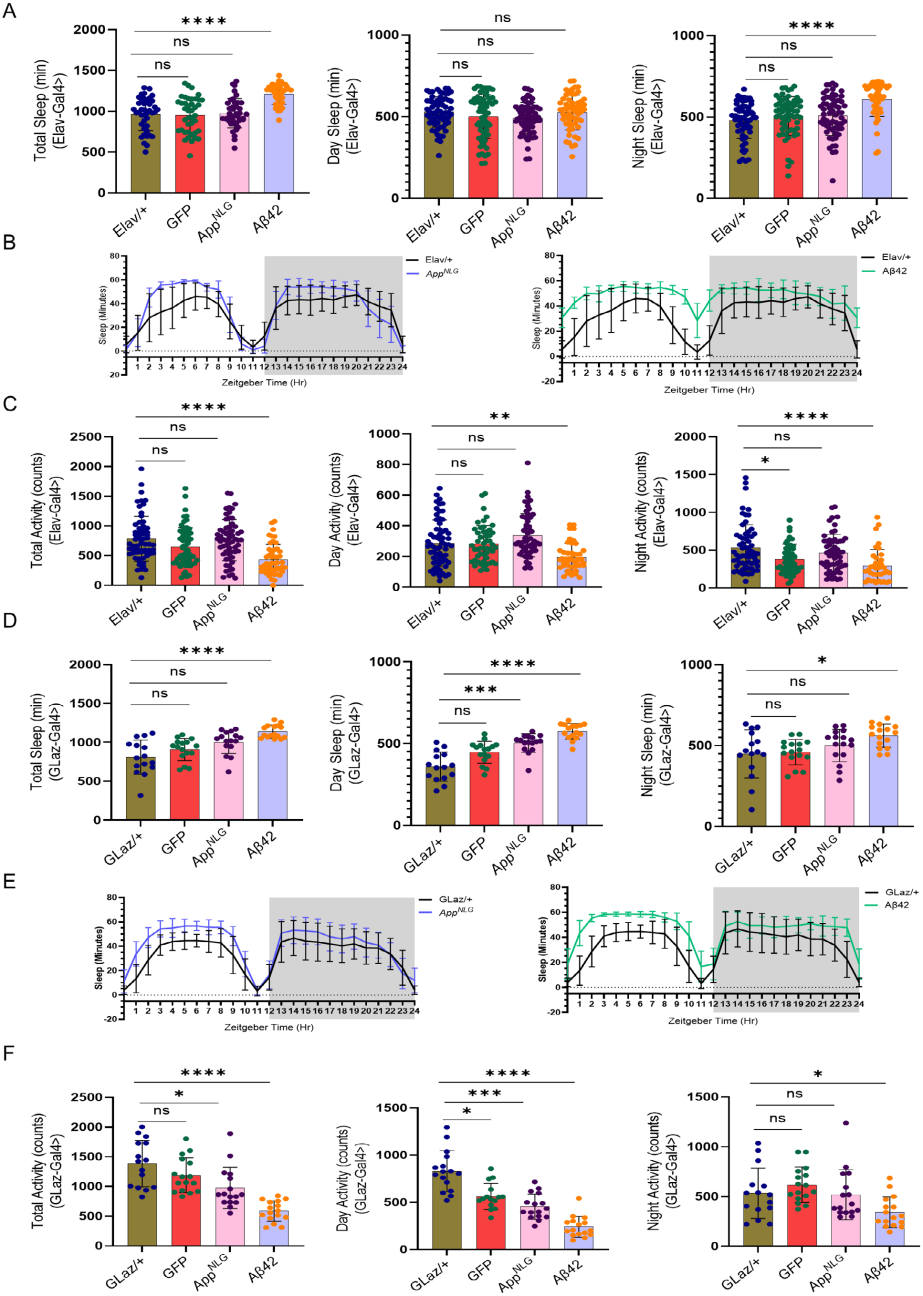
Panneuronal and glial‐specific expression of Aβ42 has compromised circadian activity and sleep quality compared to *App* ^*NLG*^, which worsens with age. (A) Total, day and night sleep (in minutes) in *Elav*‐driven *App* ^*NLG*^ and Aβ42 *m*odels. (B) Sleep profiles at different zeitgeber time (h) in *Elav*‐driven *App* ^*NLG*^ and Aβ42 models only compared to their respective controls. (C) Total, day and night sleep activity (counts) in *Elav*‐driven *App* ^*NLG*^ and Aβ42 models. (D) Total, day and night sleep (in min) in *GLaz*‐driven *App* ^*NLG*^ and Aβ42 models. (E) Sleep profiles at different zeitgeber time (h) in *GLaz*‐driven *App* ^*NLG*^ and Aβ42 models only compared to their respective controls. (F) Total, day and night sleep activity in *GLaz*‐driven *App* ^*NLG*^ and Aβ42 models. All experiments were performed in 3‐week‐old males only. Data = mean ± SD. Non‐parametric One‐way ANOVA with multiple comparisons, done with the Kruskal‐Wallis test, was performed for sleep activity and sleep fragmentation. Each dot represents the number of flies. *p < 0.05, **p < 0.01, ***p < 0.001, and ****p < 0.0001; n.s., not significant (an asterisk denotes significance for the average of all three replicates). Raw data and *p* values are provided in the source data.

Compromised circadian activity was observed in flies with panneuronal and glial‐specific expression of Aβ42, as well as in aged flies with glial‐specific expression of *App* ^*NLG*^. Various sleep and circadian parameters, including day, night, and total sleep duration, circadian activity, sleep quality, bout length, and bout number, were analyzed in 3 and 7‐week‐old male flies with panneuronal and glial‐specific expression of *App* ^*NLG*^ and Aβ42 (Figure 2A–F, Figures S1A–D and S2A–F). These data were compared to controls to evaluate the impact of AD mutations on sleep behavior. As shown in Figure 2, neuronal‐specific expression of Aβ42 led to a significant increase in overall sleep time compared to controls in 3‐week‐old flies, particularly during the night (Figure 2A). This increase in sleep was accompanied by lower sleep activity in Aβ42 (Figure 2C). Although a similar trend was observed in *App* ^*NLG*^ expressing flies, changes were not statistically significant. These findings suggest that while the panneuronal‐specific expression of Aβ42 led to more sleep, the quality of sleep was compromised due to lower sleep bout number and higher bout length (Figure S1A,B).

As shown in Figure 2D–F, Figure S1C,D, flies with glial‐specific expression of *App* ^*NLG*^ and Aβ42 exhibited significantly increased sleep, especially in those expressing Aβ42. This increase was mainly due to longer sleep during the night, as illustrated in the 24‐h sleep profile, which shows sleep min/h (Figure 2E).

This increase in sleep was associated with lower sleep activity during the day and nighttime in *App* ^*NLG*^ and Aβ42 (Figure 2F) and increased sleep bout length in both *App* ^*NLG*^ and Aβ42 (Figure S1C,D), suggesting that although total sleep duration increased, the quality of sleep was negatively impacted. The increased sleep duration was thus accompanied by poor sleep quality, as indicated by reduced activity levels.

Additionally, panneuronal expression of Aβ42 led to a decrease in the total number of sleep bouts, particularly at night, suggesting fewer but longer sleep periods (Figure S1A,B). In contrast, glial‐specific expression of *App* ^*NLG*^ and Aβ42 caused an increase in the number of sleep bouts during the day, with longer day bout lengths, indicating a distinct pattern of sleep fragmentation (Figure S1C,D). In 7‐week‐old flies, sleep disturbances persisted and worsened, indicating that these disruptions are not transient but progressively worsen with age (Figure S2A–F). Together, these findings suggest that sleep disturbances represent a persistant phenotype in AD model flies.

### 2.3 Panneuronal and Glial‐Specific Expression of *App* ^*NLG*^ and Aβ42 Led to Increased Lipid Accumulation and Reduced Synapsin Levels

**FIGURE 3:**
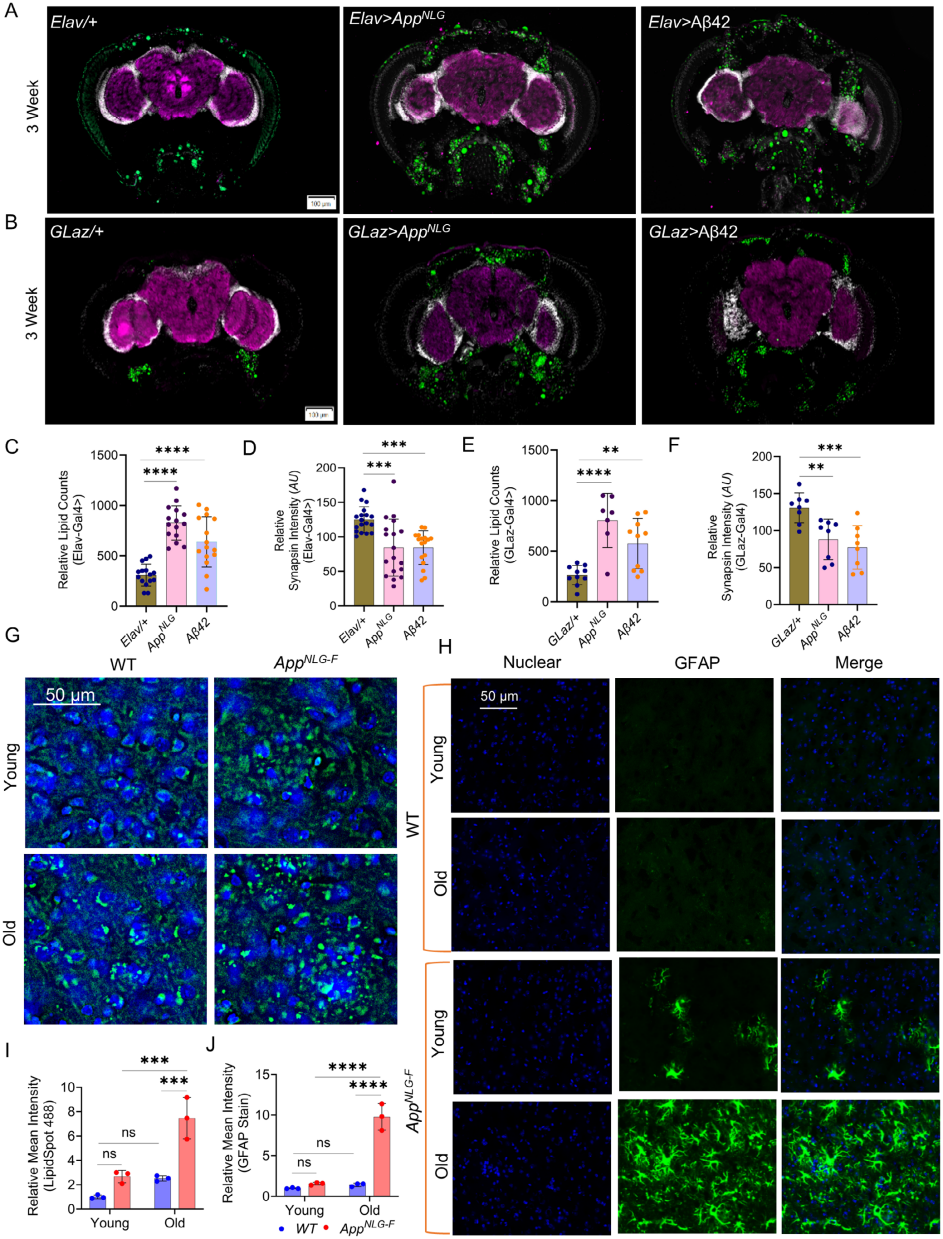
*App* ^*NLG*^ *and* Aβ42 induce lipid accumulation and synapsin reduction in *Drosophila*, while *App* ^*NLG‐F*^ mice show cortical lipid build‐up and astrocyte activation. (A, B) Representative image showing the expression of lipid accumulation (green, Lipid spot); synaptic loss (purple, anti SYNORF1), a marker of neurodegeneration; and DAPI (white), in the brains of *Elav* and *GLaz*‐driven *App* ^*NLG*^ *and* Aβ42 flies. (C–F) Quantification of the expression level of relative lipid counts (C, E) and synapsin intensity (D, F) in *Elav* and *GLaz*‐driven *App* ^*NLG*^ *and* Aβ42 flies. All experiments were performed in 3‐week‐old flies. (G) Representative images showing lipid spot intensity (green), indicating lipid accumulation; and DAPI (blue), in the mouse brains of young and old wild type (WT) and *App* ^*NLG‐F*^ models. (H) Representative images showing the immunostaining of GFAP, a marker of astrocyte activation; and DAPI (blue), in the mouse brains of young and old WT and *App* ^*NLG‐F*^ models. (I, J) Quantification of the intensity of lipid spots and relative mean intensity of GFAP immunostaining. Fold changes of fluorescence intensity were calculated relative to controls. Data = mean ± SD. One‐way ANOVA with Tukey's multiple comparisons test was performed for flies' data, with 5–6 flies per group. Two‐way ANOVA with multiple comparisons done with Uncorrected Fisher's LSD test, was performed for mouse data, with *n* = 3 mice per group. *p < 0.05, **p < 0.01, ***p < 0.001, and ****p < 0.0001; n.s., not significant (an asterisk denotes significance for the average of all three replicates). Raw data and *p* values are provided in the source data.

Our study revealed a significant increase in lipid accumulation, as indicated by LipidSpot (green) staining in both *App* ^*NLG*^ a*nd* Aβ42 flies, compared to their respective control groups at 3 weeks (Figure 3A–F). Synapsin staining intensity was significantly lower in these flies compared to controls, indicating impaired synaptic function (Figure 3D,F). This reduction was observed in both panneuronal and glial‐driven *App* ^*NLG*^ *and* Aβ42 flies. At 7 weeks, lipid accumulation remained elevated in both the *App* ^*NLG*^ a*nd* Aβ42 flies in both panneuronal and glial drivers. Synapsin intensity was unchanged in panneuronal‐driven flies but was reduced in the glial‐driven *App* ^*NLG*^ model, suggesting a stabilization or compensatory adaptation in synaptic function (Figure S3D,F). These findings indicate an early phase of synaptic damage followed by a potential plateau in synaptic decline, with persistent lipid and amyloid changes pointing to ongoing pathophysiological processes. Overall, the results suggest that lipid dysregulation and synapsin impairment are associated in AD progression, with the *App* ^*NLG*^ model potentially reflecting more closely the synaptic dysfunction observed in AD, which may contribute to cognitive and sleep disturbances. These results are also consistent with the concept that small, soluble Aβ oligomers are highly synaptotoxic and can interact with neuronal membranes, whereas larger aggregated assemblies are comparatively less effective at disrupting synaptic function and cannot penetrate the membranes, providing a plausible link between lipid dysregulation, synaptic impairment, and downstream glial activation (Butterfield et al. 2007; Mattson 2004; Walsh et al. 2002; Walsh and Selkoe 2004). This oligomer‐membrane interaction triggers oxidative stress, calcium dysregulation, and ultimately neuronal death, while also activating glial cells and initiating inflammatory responses (Butterfield and Lauderback 2002; Mattson 2007).

### 2.4 *App* ^*NLG*^ ^*‐F*^ Mice Exhibit an Age‐Dependent Increase in Lipid Accumulation and Glial Activation

We performed immunostaining in the cortex (Ctx) region of the *App* ^*NLG‐F*^ mice to assess LipidSpot staining and GFAP immunoreactivity at different ages (young and old mice). In 15‐month‐old *App* ^*NLG‐F*^ mice, we observed a notable increase in lipid accumulation, as indicated by enhanced LipidSpot intensity (Figure 3G,I). Additionally, GFAP level, which marks astrocytic activation, was significantly higher in 15‐month‐old *App* ^*NLG‐F*^ compared to age‐matched wild‐type (WT) controls (Figure 3H,J).

**FIGURE 4:**
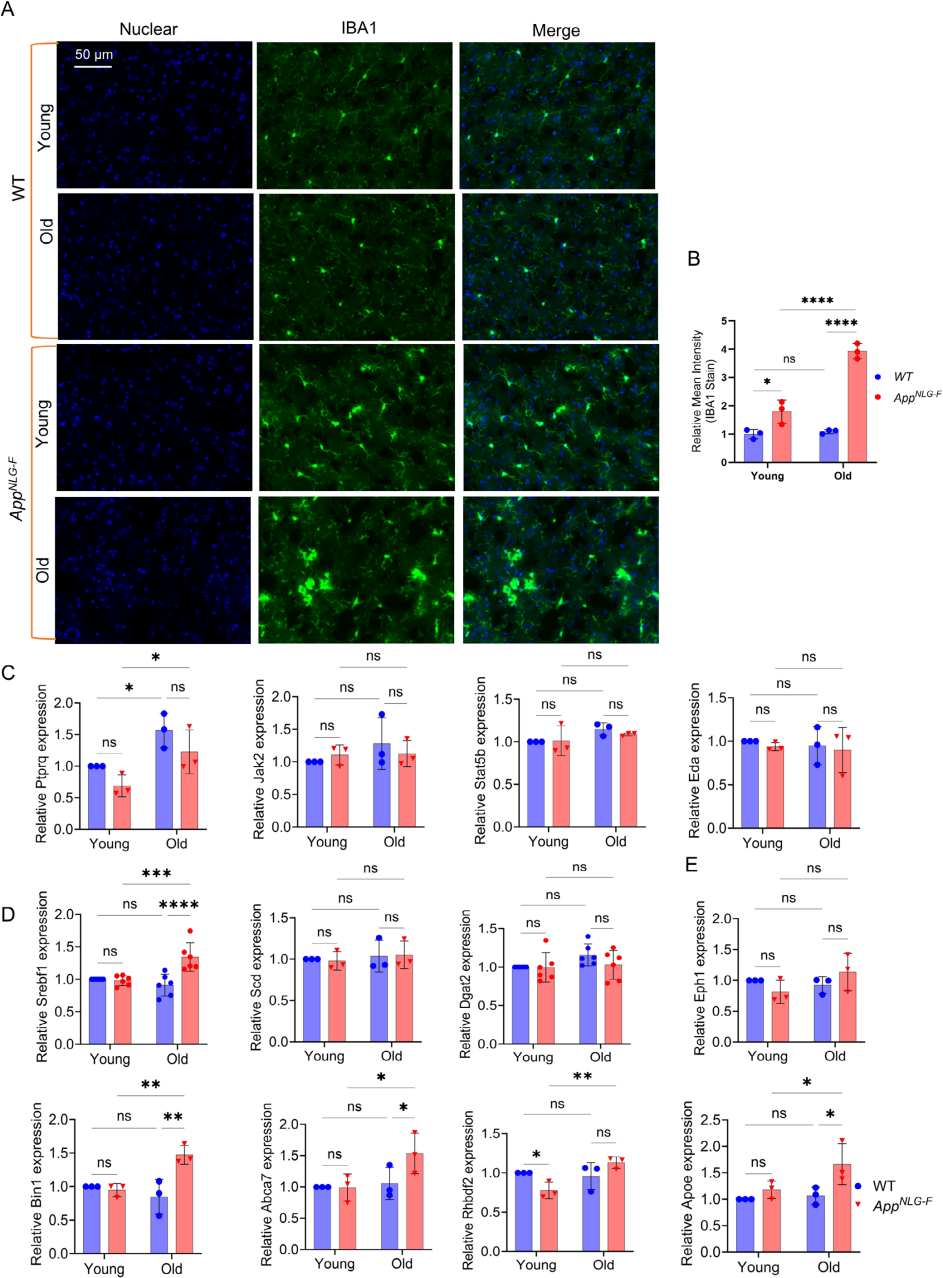
Age‐dependent increase in neuroinflammation and AD risk gene expression in a mouse model of *App* ^*NLG‐F*^. (A) Immunofluorescence staining of IBA1 protein in microglia within the cortex region of the brain of WT and *App* ^*NLG‐F*^ mice at young and old age. (B) Quantification of the IBA1 immunostaining intensity in (A); *n* = 3 mice per group. (C–E) Quantitative PCR quantification of the relative expression level of inflammatory genes, *Ptprq*, *Jak2*, *Eda*, *and Stat5b* (C), metabolic genes, *srebf1*, *scd*, and *Dgat2* (D), and AD risk genes, *Bin1*, *Abca7*, *Rhbdf2*, and *Apoe* (E), in young and old WT and *App* ^*NLG‐F*^ mice. Data = mean ± SD. Two‐way ANOVA with multiple comparisons, done with Uncorrected Fisher's LSD test, was performed. *p < 0.05, **p < 0.01, ***p < 0.001, and ****p < 0.0001; n.s., not significant (an asterisk denotes significance for the average of all three replicates). Raw data and *p* values are provided in the source data.

We also examined Nile red and DGAT2 staining in the Ctx (Figure S3G–J). Nile red staining significantly increased in old *App* ^*NLG‐F*^ mice, while DGAT2 staining showed no change in intensity, although DGAT2 aggregates were observed in the hippocampal (CA1) region and cerebellum (Figure S4A–D). Furthermore, we evaluated Adipose differentiation related protein (ADRP), a protein that binds lipid droplets, and found no significant changes in its levels in the Ctx or CA1 regions (Figure S5A–D). Immunostaining for GFAP and IBA1 in the CA1 region (Figure S6A–D) revealed a significant increase in GFAP‐positive cells in 15‐month‐old *App* ^*NLG‐F*^ mice. IBA1‐positive cells, marking microglial activation, were also significantly higher in both young and old *App* ^*NLG‐F*^ mice compared to WT controls. Notably, aging did not affect IBA1 expression in WT mice, but a dramatic increase in IBA1 was observed in 15‐month‐old *App* ^*NLG‐F*^ mice, both in the CA1 region (Figure S6B,D) and in the Ctx (Figure 4A,B). Together, these findings suggest that aging in *App* ^*NLG‐F*^ mice is associated with increased lipid accumulation, astrocytic activation, and microglial changes, consistent with age‐related neuroinflammation in AD models.

### 2.5 Age‐Dependent Increase in AD Risk Gene Expression in the *App* ^*NLG‐F*^ Mouse Model

In the *App* ^*NLG‐F*^ mice, we observed a significant age‐dependent increase in IBA1, a marker of microglia activation (Figure 4A,B, Figure S6B,D). This rise in IBA1 immunoreactivity suggests that microglia become progressively activated with age, possibly in response to progressive amyloid pathology. Microglia initially respond to plaques and other damage by becoming activated to clear harmful debris, but chronic activation over time can lead to neuroinflammation. This persistent activation may reduce the efficiency of microglial function, potentially contributing to neuronal damage and the progression of neurodegenerative diseases like Alzheimer's. To explore this further, we examined the expression of key inflammatory genes. Notably, protein tyrosine phosphatase receptor type Q (*Ptprq*) showed a marked age dependent upregulation (Figure 4C), supporting the idea of chronic, age‐related inflammation. However, other inflammatory markers, such as Janus kinase2 (*Jak2*), ectodysplasin (*Eda*), and signal transducer and activator of transcription 5B (*Stat5b*) did not show significant change, suggesting a more selective inflammatory response. In addition to neuroinflammation, we assessed the expression of metabolic genes. Notably, sterol regulatory element binding protein (Srebf1) expression was significantly increased with age, while no significant changes were observed in other metabolic genes, such as stearoyl‐CoA desaturase (*Scd*) and *Dgat2*, indicating a possible alteration in lipid metabolism (Figure 4D). Further investigation of AD risk genes revealed an age‐dependent increase in the expression of Bridging integrator 1 (*Bin1*) and ATP binding cassette subfamily A member 7 (*Abca7*), both of which are involved in amyloid processing and lipid transport (Figure 4E). This upregulation suggests a potential link to increased amyloid burden or alterations in lipid metabolism, both of which are key features of Alzheimer's pathology. Additionally, we observed elevated expression of rhomboid family‐like 2 (*Rhbdf2*) and Apoe genes that are also associated with neuroinflammation and lipid homeostasis (Figure 4E), and the age‐dependent increases of *Bin1* and *Abca7* are only shown in AD models but not in WT mice.

Taken together, the increased expression of IBA1, *Ptprq*, and several AD risk genes with age highlights the progressive activation of neuroinflammatory pathways and suggests that lipid metabolism and inflammatory responses are central contributors to the neurodegenerative processes in the *App* ^*NLG‐F*^ mouse model. This growing evidence reinforces the idea that neuroinflammation and the dysregulation of AD‐related pathways progressively exacerbate pathology as the mice age.

### 2.6 *App* ^*NLG*^ and Aβ42‐Linked Locomotor and Cognitive Dysfunction Were Improved With *Dgat2* *Knockdown*

**FIGURE 5:**
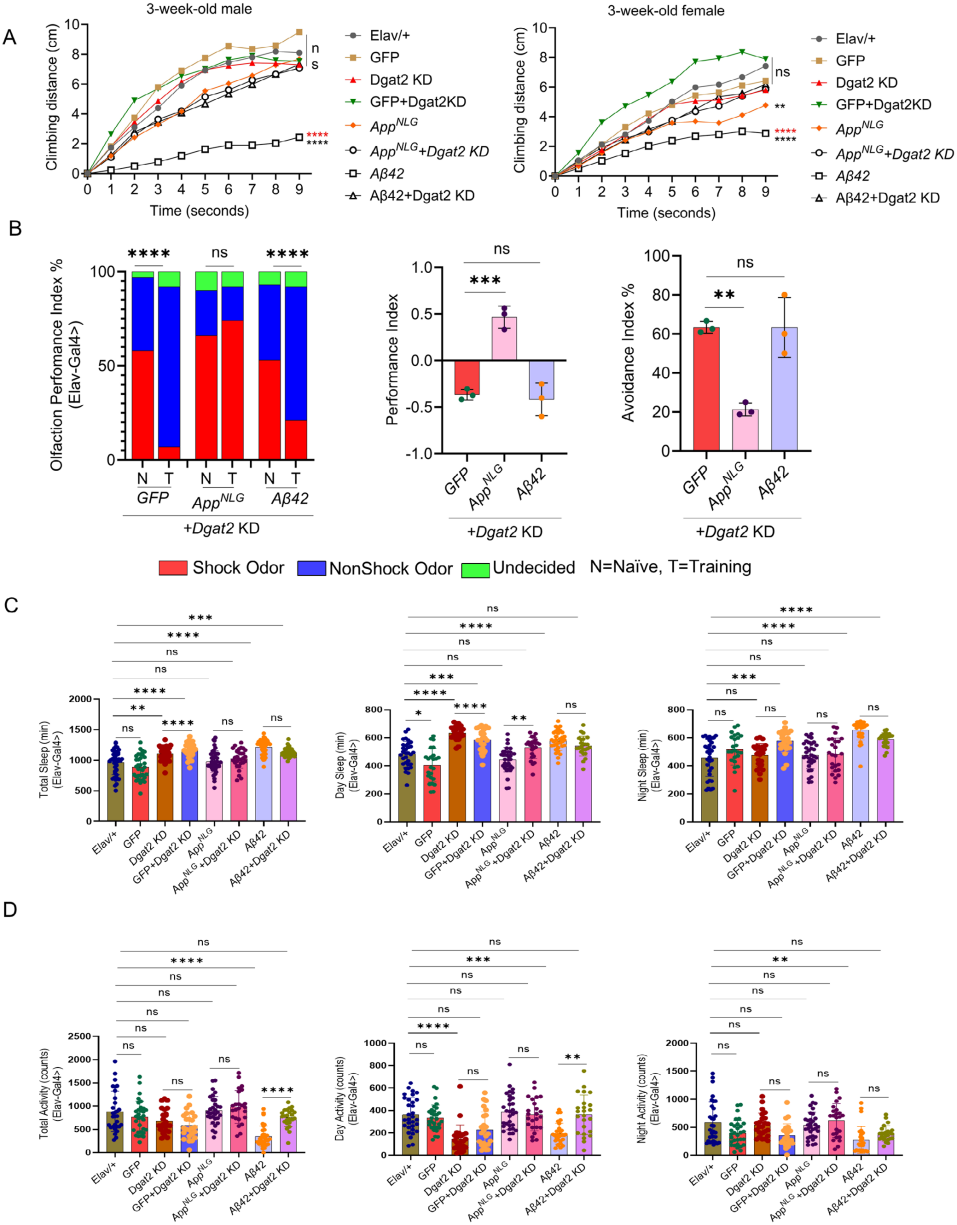
*Dgat2* *KD* enhances locomotor and cognitive performance, restores sleep activity, and reduces fragmentation linked to *App* ^*NLG*^ *and* Aβ42 expression. (A) Negative geotaxis assay of *Elav*‐driven *Dgat2* *KD* in A*pp* ^*NLG*^ *and* Aβ42 models in 3‐week male and female (red asterisk denotes significance with Aβ42 *+ Dgat2* *KD* and/or *App* ^*NLG*^ *+Dgat2* *KD* while black asterisks, ns, denotes significance with *Elav/+*). (B, left panels only) *Dgat2* *KD* overcame deficits in olfactory aversion training in *Elav*‐driven 3‐week‐old male *App* ^*NLG*^ *and* Aβ42 models; data combined from triplicate assays. The significance was based on the chi‐square test. Values are shown as the percentage of flies in each chamber at the end of the assay. (B, middle panels) the difference between fly decisions in *Elav*‐driven *Dgat2* *KD* in 3‐week‐old male *App* ^*NLG*^ *and Aβ42* models. (B, right panels), flies avoiding shock odor in *Elav*‐driven *Dgat2* *KD* in 3‐week‐old male *App* ^*NLG*^ *and* Aβ42 models. (C) Total, day and night sleep (in min) in *Elav*‐driven *Dgat2* *KD* in *App* ^*NLG*^ *and* Aβ42 models. (D) Total, day and night sleep activity (in counts) in *Elav*‐driven *Dgat2* *KD* in *App* ^*NLG*^ *and Aβ42* models. Data = mean ± SD. One‐way ANOVA with Tukey's multiple comparisons test was performed (Left and right panels only). Two‐way ANOVA with Tukey's multiple comparisons test was performed for geotaxis, *n* = 3 replicates per group, and each group had 10 flies. Non‐parametric One‐way ANOVA with multiple comparisons, done with the Kruskal‐Wallis test, was performed for sleep activity and sleep fragmentation. *p < 0.05, **p < 0.01, ***p < 0.001, and ****p < 0.0001; n.s., not significant (an asterisk denotes significance for the average of all three replicates). Raw data and *p* values are provided in the source data.

We assessed geotaxis and cognitive function in 3‐week‐old flies to investigate the effects of *App* ^*NLG*^ and Aβ42 on the panneuronal *Dgat2* *KD*. First, a negative geotaxis assay was used to assess locomotor performance. At 3 weeks of age, significant differences in locomotor performance were observed between flies expressing the *App* ^*NLG*^ gene and their respective controls. Male flies with panneuronal *Dgat2* *KD* displayed improved climbing ability when expressing *App* ^*NLG*^ *or* Aβ42, *w*hile female flies showed similar trends, though less significantly (Figure 5A). These results suggest that *Dgat2* *KD* may partially rescue locomotor deficiets associated with AD‐linked gene expression.

We next evaluated whether cognitive performance was altered following *Dgat* *KD*. Male flies expressing GFP, Aβ42, and *App* ^*NLG*^ exhibited memory impairment, which was rescued by *Dgat2* *KD* (Figure 5B, left panel). Further analysis of odor avoidance using the performance index (Figure 1C–E) supported these findings, reinforcing the idea that *Dgat2* modulation affects both locomotion and cognition (Figure 5B,C, middle and right panel). In conclusion, our results suggest that *Dgat2* *KD* has a protective effect, improving both locomotor and cognitive function in these areas.

### 2.7 *Dgat2* *Knockdown* Leads to the Restoration of Sleep Activity Linked With the Expression of *App* ^*NLG*^ and Aβ42

We assessed sleep activity in *Elav*‐driven *Dgat2* *KD* and at both 3 and 7 weeks of age to explore the potential links between sleep, cognition, and memory performance, which were previously observed in *App* ^*NLG*^ and Aβ42. At 3 weeks of age, *Dgat2* *KD* flies expressing Aβ42 exhibited a more prominent increase in total sleep, including daytime and nighttime sleep compared to *App* ^*NLG*^. *Dgat2* *KD* showed increased sleep timing, indicating a more continuous sleep pattern (Figure 5C,D). These suggest that *Dgat2* manipulation at 3 weeks drastically restores the sleep pattern of *App* ^*NLG*^ and Aβ42. Further investigation revealed changes in sleep activity in the *App* ^*NLG*^ and Aβ42 models, such as fewer sleep bouts and increased bout length, particularly in flies with *Elav*‐driven *Dgat2* *KD* (Figure S9). However, at 7 weeks, sleep behavior in *App* ^*NLG*^ and Aβ42 models with *Dgat2* *KD* was largely unchanged compared to the 3‐week‐old flies (Figure S9A–D). This suggests that the impact of *Dgat2* manipulation on sleep may be age‐dependent, with limited additional changes observed in older flies.

### 2.8 *Dgat2* *Knockdown* Suppressed Lipid Metabolism, Restored Synaptic Loss, and Reduced Inflammation Associated With *App* ^*NLG*^ and Aβ42

**FIGURE 6:**
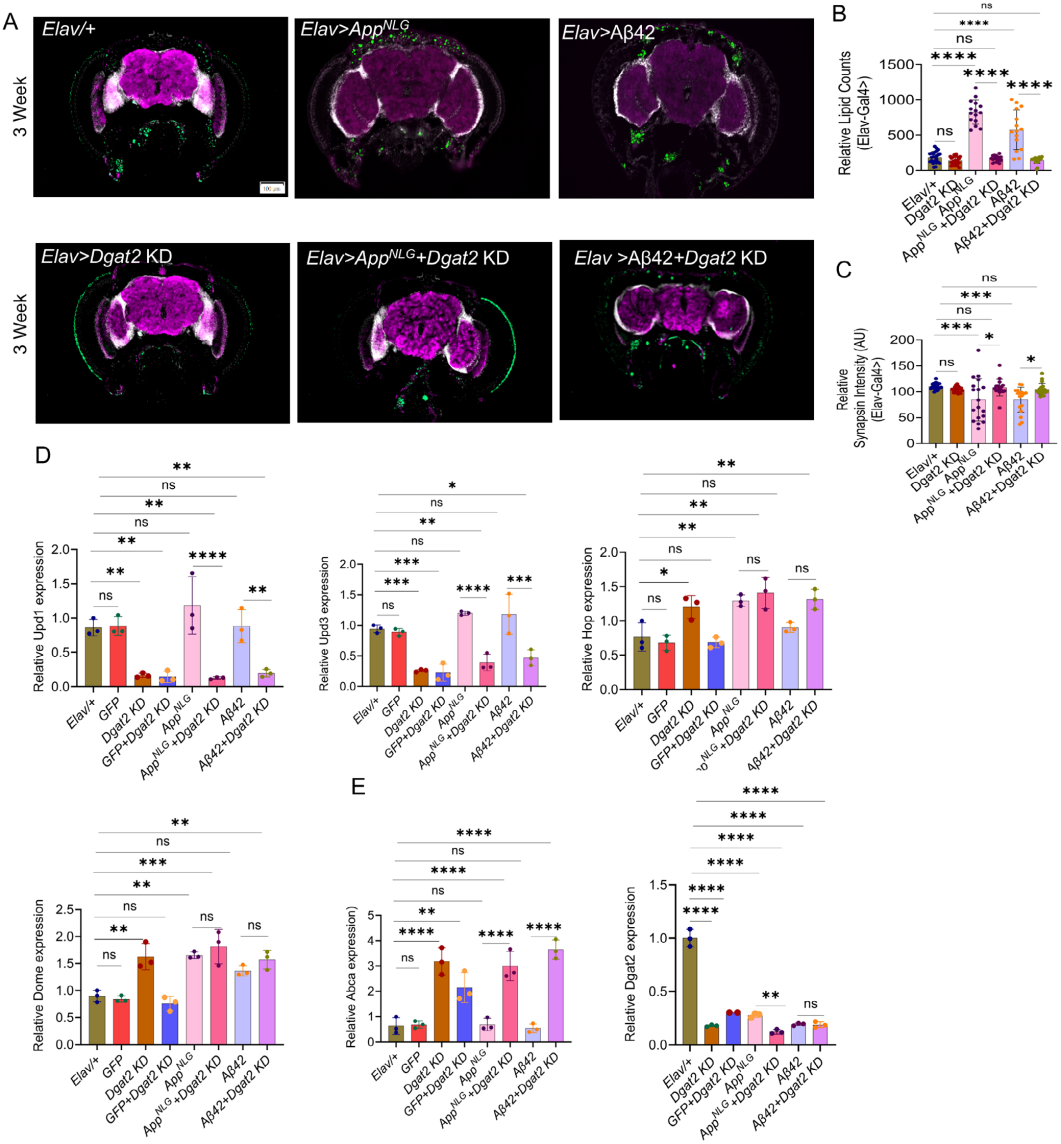
*Dgat2* *KD* suppresses lipid metabolism, restores synaptic loss, and reduces inflammation in *App* ^*NLG*^ *and* Aβ42 models of *Drosophila*. (A) Representative image showing the expression of lipid accumulation (green, Lipid spot 488); synaptic loss (purple, anti SYNORF1); a marker of neurodegeneration, and DAPI (white), in the brains of *Elav*‐driven *KD* in *App* ^*NLG*^ *and* Aβ42 models. (B, C) Quantification of the expression level of lipid counts and synapsin intensity in *Elav*‐driven *App* ^*NLG*^ and Aβ42 models. (D) Relative expression of inflammatory genes, *Upd1*, *Upd3*, *Hop* and *Dome* in flies. (E) Relative expression of metabolic genes, *Abca* and *Dgat2* in flies. Data = mean ± SD. One‐way ANOVA with Tukey's multiple comparisons test was performed . *n* = 3 replicates per group, and each group has 8–10 flies. *p < 0.05, **p < 0.01, ***p < 0.001, and ****p < 0.0001; n.s., not significant (an asterisk denotes significance for the average of all three replicates). Raw data and *p* values are provided in the source data.

We assessed immunostaining in *Elav*‐driven *Dgat2* *KD* flies expressing *App* ^*NLG*^ *and* Aβ42 at 3 weeks to examine potential changes in lipid metabolism and synaptic function. At this early age, we observed that lipid accumulation, typically elevated in *App* ^*NLG*^ and Aβ42 models, was significantly reduced in the presence of *Dgat2* *KD*. This suggests that *Dgat2* *KD* mitigates abnormal lipid buildup associated with AD models. Interestingly, synapsin levels were notably elevated in these flies (Figure 6A–C), indicating improved synaptic function in response to decreased lipid accumulation. At 7 weeks, lipid levels remained reduced in the presence of *Dgat2* *KD*, suggesting sustained mitigation of lipid dysregulation. However, synapsin levels were unaffected at 7 weeks (Figure S10A–C), indicating that neither the expression of the *App^NLG^* or Aβ42 nor the continued reduction in lipid accumulation due to *Dgat2* *KD*, synapsin levels remain unchanged at this age. This suggests that *Dgat2* *KD* has a persistent effect on lipid metabolism, but its impact on synaptic function may be age dependent.

We further examined neuroinflammatory markers. The levels of primary inflammatory cytokines, *unpaired1* (*Upd1*) and *unpaired3* (*Upd3*), were significantly reduced in *Dgat2* *KD* flies, indicating decreased neuroinflammation. However, expression of the JAK/STAT pathway receptors, *domeless* (*Dome*) and *Hopscotch* (*Hop*), was increased, suggesting that the reduction in inflammation may activate compensatory signaling through this pathway (Figure 6D). We also analyzed the expression of metabolic markers *Abca* and *Dgat2*. In both *App* ^*NLG*^ and *Aβ42* models, *Dgat2* *KD* led to a marked reduction in *Dgat2* expression, confirming the efficiency of the *KD* and validating its use in these neurodegenerative backgrounds. At the same time, we observed a consistent increase in *Abca* expression under these conditions. This reciprocal pattern suggests that loss of *Dgat2*, an enzyme central to triglyceride synthesis and lipid droplet formation, perturbs lipid metabolism and elicits a compensatory upregulation of *Abca*. Together, these findings indicate that *Dgat2* downregulation disrupts lipid storage pathways, while Abca upregulation may act to maintain lipid homeostasis, underscoring a critical interplay between lipid synthesis and transport in neurodegeneration (Figure 6E).

**FIGURE 7:**
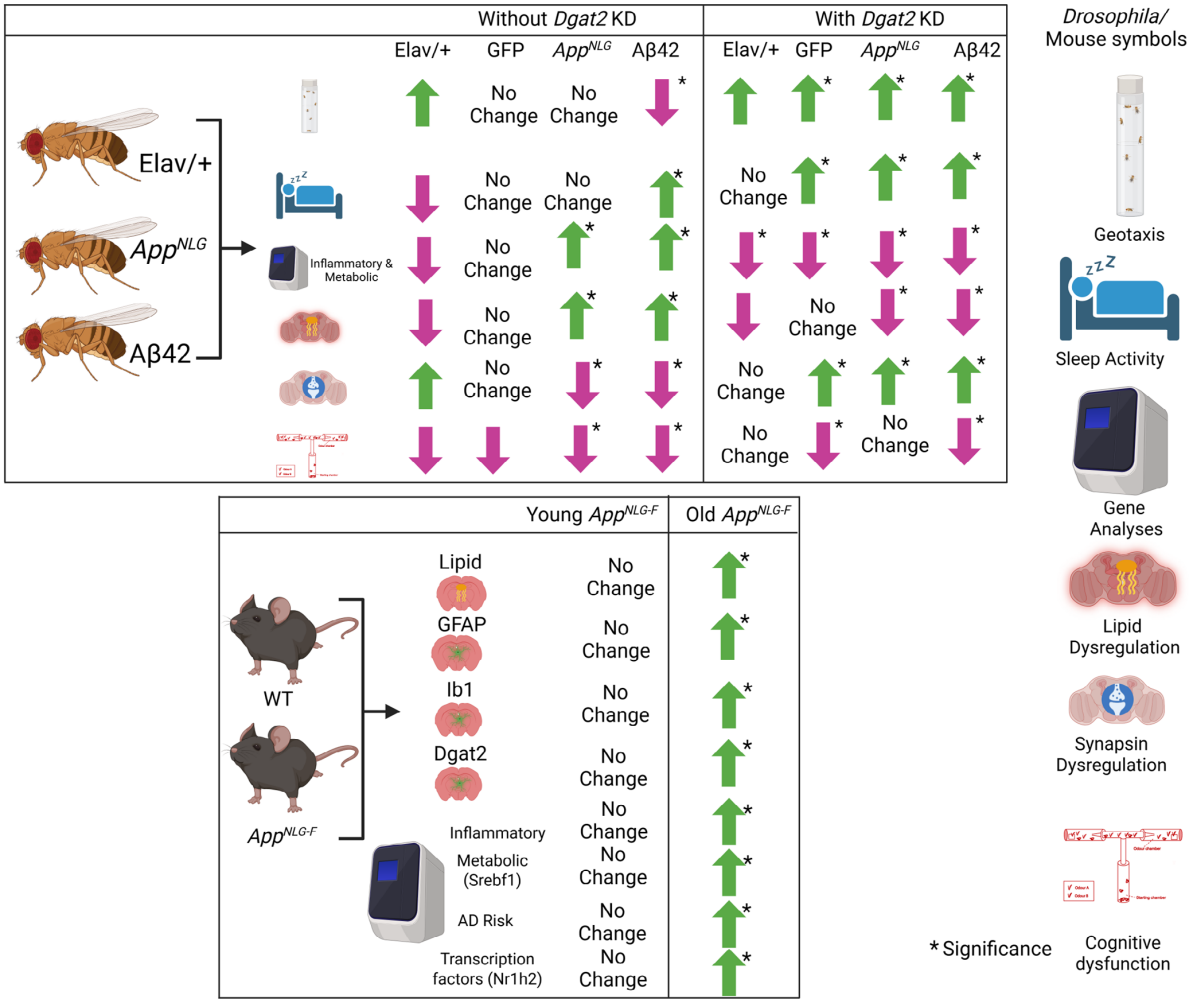
Graphical illustration showed the impacts of panneuronal and glial‐specific expression of *App* ^*NLG*^ *and* Aβ42 without and with *Dgat2* *KD* in *Drosophila* and microglial/astrocytic activation and lipid accumulation in *App* ^*NLG‐F*^ mice. The top panel result shows that panneuronal and glial‐specific expression of *App* ^*NLG*^ *and* Aβ42 led to compromised locomotor and cognitive performance. It also led to enhanced lipid accumulation and reduced synapsin levels, which were further rescued with *Dgat2* *KD* (up and down arrows). The bottom panel shows the changes in GFAP, IBA1, *Dgat2* stain, inflammatory, and AD risk gene expression in young/old *App* ^*NLG‐F*^ mice.

Overall, we have shown a summary of the behavior, physiological, and cytological alterations with the expression of AD‐linked genes in *Drosophila* and mouse models. Moreover, the impact of *Dgat2* on modulating AD phenotype has also been summarized in Figure 7. These findings underscore the intricate connection between amyloid pathology, lipid dysregulation, and neuroinflammation, suggesting that targeting *Dgat2*, through conserved lipid homeostasis mechanisms across species, may offer a novel and translationally relevant therapeutic approach for Alzheimer's disease.

## 3 Discussion

Alzheimer's disease (AD) is characterized by accumulation of soluble Aβ oligomers and aggregated amyloid deposits, along with tau neurofibrillary tangles, leading to cognitive decline and neurodegeneration through synaptotoxicity and neural dysfunction (Zhang et al. 2021). Importantly, soluble Aβ oligomers, compared with insoluble aggregates, show a strong association with synapse loss and cognitive decline in AD patients (Hong et al. 2016; Shankar et al. 2008). Genetic models, such as *Drosophila* expressing APP mutations, offer valuable insights into the mechanisms underlying AD (Padmanabhan and Gotz 2023). Here we investigated the effects of *App* ^*NLG*^, as well as the aggregation‐prone Aβ42 on locomotor function, memory, and lipid metabolism in *Drosophila*. Additionally, we assessed the role of Dgat2, a key enzyme in lipid metabolism, through its *KD* (Rong et al. 2024). To further strengthen our findings, we used the *App* ^*NLG‐F*^ mouse model of AD, allowing for cross‐species comparison of lipid profiles, neuroinflammation, and gene expression. Through locomotor assays, cognitive tests, lipid analysis, and gene expression studies, we sought to determine how these genetic modifications influence AD pathogenesis and identify potential therapeutic strategies for mitigating AD‐associated dysfunction.

Here, we have used *Drosophila* and *mouse* models of AD to investigate the effect of APP and Aβ42 with and without *Dgat2* modulation (*Drosophila*) and with young and old (Mouse) on AD‐related phenotypes including synaptic dysfunction. Panneuronal, glial, and mushroom body‐specific expression of UAS*‐App* ^*NLG*^, UAS‐Aβ42 induced lipid dysmetabolism and neurodegeneration in flies. Our data in *Drosophila* models of *App* ^*NLG*^ and Aβ42 showed progressive locomotor impairments, sleep‐circadian disturbances, memory deficits, lipid accumulation, synaptic loss, and neuroinflammation. *Knockdown* of *Dgat2* improved inflammatory and metabolic gene expression, enhanced locomotor performance, and modulated sleep activity and circadian rhythms, suggesting that modulation of lipid metabolism via Dgat*2* may confers protective effects. In mice, we used *App* ^*NLG‐F*^ knock‐in animals with elevated pathogenic Aβ and oligomer levels due to the combined effects of three mutations associated with familial AD (Yokoyama et al. 2022). These mice developed oligomeric Aβ species, amyloid deposits, neuroinflammation, synaptic loss, and cognitive deficits, providing a robust model to study the AD progression. In this model, lipid accumulation and neuroinflammation were observed. These findings suggest that targeting Dgat2 may offer a potential therapeutic strategy for AD, emphasizing the conserved impact of lipid metabolism across species and its potential role in modulating oligomer‐mediated toxicity.

Our findings highlight the complex interplay among oligomeric amyloid pathology, lipid metabolism, and neuroinflammation across species using *Drosophila* and mouse AD models. We found that male *Drosophila* expressing Aβ42 exhibited significant locomotor impairments, particularly reduced climbing ability in the geotaxis assay. Interestingly, the decline was more pronounced with panneuronal or glial‐specific expression of Aβ42 compared with flies expressing *App* ^*NLG*^ construct, suggesting that the geotaxis phenotype is mutation‐specific (Figure 1B). This is consistent with previous studies linking amyloid‐related motor deficits to the expression of toxic *Aβ* species (Sun et al. 2022). Cognitive assays also revealed significant memory impairment, particularly in flies expressing Aβ42 in the mushroom bodies, a key brain region critical for olfactory learning (Figure 1C–E). This observation underscores the critical role of specific brain regions in cognitive dysfunction in AD, consistent with findings in mammalian models where oligomeric amyloid pathology in the hippocampus and cortex impairs memory through synaptotoxicity (Polis and Samson 2024; Selkoe 2008; Shankar et al. 2008).

Our findings showed significant sleep disruptions in *Drosophila* models expressing Aβ42, including increased sleep duration and altered sleep architecture. These disruptions were more severe compared to the APP mutation model, indicating that the Aβ42 expression has a stronger impact on sleep quality. The increase in total sleep time, predominantly during the nighttime (Figure 2A,B), aligns with previous research showing that AD models often exhibit prolonged sleep durations, especially at night (Insel et al. 2021; Liu et al. 2021). However, our results also showed a decrease in activity during sleep periods (in counts), particularly in the *Elav*‐ and *GLaz*‐driven models, suggesting that while these flies sleep more, sleep quality is reduced (Figure 2A–F). Furthermore, decreased activity levels, particularly in the *Elav*‐driven model, suggested that the sleep changes were closely tied to disruptions in overall activity and circadian rhythms, as seen in both *Drosophila* and mammalian AD models (Duncan et al. 2022; Majcin Dorcikova et al. 2023; Winer et al. 2021). The persistence of sleep disturbances in older flies suggested that sleep dysregulation was a chronic feature of AD pathology, much like the long‐term sleep issues observed in AD patients (Bellesi et al. 2017; Minakawa et al. 2017). The differences in sleep fragmentation patterns between the neuronal and glial‐specific models highlight the potential role of glial cells in modulating sleep, with glial‐specific expression leading to a distinct pattern of increased daytime sleep bouts and longer bout lengths. This is consistent with recent studies implicating glial activation in the disruption of sleep architecture in AD models (Bellesi et al. 2017; Garofalo et al. 2020; Gentry et al. 2022). Collectively, these results reinforce the idea that sleep disturbances, particularly fragmented sleep, are a central feature of AD pathology and may serve as an early indicator of disease progression.

Our data are consistent with the notion that lipid dysregulation and synaptic impairment play crucial roles in the progression of AD, with distinct differences in the severity of these alterations between the Aβ42 *and App* ^*NLG*^ models. Membrane lipid composition may affect oligomer assembly and membrane interactions, contributing to the lipid deposits observed in AD pathology. Lipid accumulation was significantly elevated in both *Drosophila* and mouse models of AD, particularly in the Aβ42 model, which exhibited more pronounced lipid deposition compared to the *App* ^*NLG*^ model (Figure 3A–F). This aligns with previous research indicating that dysregulated lipid metabolism, particularly the accumulation of lipid droplets, is a key feature of AD pathogenesis (Liu et al. 2021). Additionally, alterations in membrane lipid compositions, including changes in cholesterol, sphingomyelin, and gangliosides, can promote oligomer formation and enhance neurotoxic effects (Matsuzaki 2014; Numaguchi et al. 2024). Such imbalances may contribute to cellular stress and dysfunction, particularly in neurons and glial cells, and potentially exacerbate the cognitive and behavioral deficits seen in AD (Fan et al. 2020; Keeney et al. 2013). Beyond accumulation of lipid droplets, AD entails specific membrane phospholipid losses, specifically PtdEtn plasmalogens, which decrease antioxidant capacity and induce susceptibility to lipid peroxidation (Dorninger et al. 2020; Wood et al. 2015). The *App* ^*NLG‐F*^ model reproduces these age‐dependent phospholipid alterations (Emre et al. 2021). These alterations in membrane compositions may drive neuroinflammation through several mechanisms, including generation of reactive lipid species that activate glial cells (Butterfield et al. 2007), disruption of lipid asymmetry that elicits microglial responses, and promotion of reactive astrocyte formation via lipid‐mediated signaling (Joshi et al. 2019; Liddelow et al. 2017). Consequently, the observed inflammation and lipid accumulation may reflect a fundamental membrane phospholipid dysregulation, which could propagate oligomer toxicity and accelerate neurodegenerative process.

In addition to lipid accumulation, we found a marked reduction in synapsin expression, a protein essential for synaptic function (Figure 3D,F). This finding is consistent with previous studies showing that synaptic loss and dysfunction are hallmarks of AD and are often correlated with cognitive decline driven by toxic Aβ species (Ferreira et al. 2015; Lan et al. 2022; Saroja et al. 2022). The more pronounced reductions in synapsin in the Aβ42 model suggest that Aβ may exert a stronger synaptotoxic effect than APP mutation based models such as *App^NLG^* (Piccoli et al. 2022). Transitioning to the *App* ^*NLG‐F*^ mouse model further corroborates these findings, showing a significant increase in lipid accumulation and glial activation, particularly with aging, suggesting that lipid dysregulation and neuroinflammation are progressive features of AD pathology (Figure 3G–J, Figures S3G–J, S4, and S5). Aging fuexacerbates lipid accumulation and astrocyte activation, a key aspect of AD progression (Emre et al. 2021). Elevated GFAP level, indicative of glial activation, further supports the idea that lipid imbalances are coupled with neuroinflammation in the aging brain (Schuitemaker et al. 2012).

Microglial activation and neuroinflammation played pivotal roles in AD progression, as evidenced by increased IBA1, a marker for microglial activation, in the *App* ^*NLG‐F*^ mouse model (Figure 4A,B). This age‐dependent increase in IBA1 suggests that chronic neuroinflammation became more pronounced with advancing age, consistent with studies linking microglial activation to neurodegenerative diseases, including AD (Keeney et al. 2013). The increase in IBA1 aligns with reports indicating that sustained microglial activation is a driver of neurodegeneration (Agrawal and Jha 2020; Sakuraba et al. 2013). Furthermore, the observed upregulation of *Ptprq* (Figure 4c) corroborates the presence of chronic, age‐related inflammation in these mice, consistent with other reports on the role of neuroinflammation in AD pathology (Prendecki et al. 2020). Interestingly, the increased expression of key AD risk genes, including *Bin1*, *Rhbdf2*, *Apoe*, and *Abca7*, which are involved in amyloid processing and lipid metabolism (Figure 4E), suggests that oligomeric Aβ burden or lipid dysmetabolism worsen with age, further implicating lipid pathways in AD progression (De Jager et al. 2014). Furthermore, the elevation of *Rhbdf2* and *ApoE*, both of which are linked to neuroinflammation and lipid homeostasis, underscores the role for disrupted lipid pathways in AD risk (Stone et al. 2009). Collectively, these findings point to a progressive activation of inflammatory and amyloid‐related pathways in the *App* ^*NLG‐F*^ model, with aging exacerbating neuroinflammatory responses. Our findings are also aligned with recent lipidomic studies, such as the *neurolipid atlas*, which revealed that ApoE4‐driven cholesterol ester accumulation in astrocytes is associated with impaired immune signaling and antigen presentation pathways (Feringa et al. 2025). Overall, these findings support the concept that lipid dysregulation and neuroinflammation are mechanistically connected in AD, connected with our results across *Drosophila* and mouse models. The combination of these factors highlights the importance of neuroinflammation in driving disease progression in AD and suggests potential therapeutic avenues targeting these pathways.

Our study also established how panneuronal *KD* of *Dgat2* influences lipid metabolism and neurodegeneration associated with *Drosophila* AD models. Dgat2, a key enzyme involved in lipid biosynthesis, has been implicated in neuroinflammation and neurodegenerative processes (Kang and Rivest 2012; Moraes et al. 2024). *Dgat2* *KD* in *Drosophila* models indicated that lipid metabolism plays a critical role in locomotor, cognitive, and sleep deficits associated with AD. *Dgat2* *KD* improved locomotor function, specifically in male flies expressing APP‐mutant genes (*App* ^*NLG*^ *and* Aβ42), where *KD* partially rescued climbing ability (Figure 5A). This suggests that *Dgat2* *KD* may mitigate detrimental effects associated with oligomeric Aβ42 and aggregation and provide protection against motor impairments seen in AD (Broussard and Brady 2010). *Dgat2* *KD* protective effects may extend beyond reducing triglycerol accumulation by preserving PtdEtn, membrane phospholipid composition and bilayer asymmetry (Dorninger et al. 2020; Emre et al. 2021). These alterations may reduce inflammatory signaling by limiting lipid peroxidation, inhibiting phosphatidylserine externalization that activates microglia, and decreasing oligomer membrane insertion (Chew et al. 2020). This multi‐level membrane protection may explain the extensive anti‐inflammatory effects and enhanced synaptic markers observed.

Cognitive function assessed via the olfactory aversion memory assay, mirrored the locomotor findings. *Dgat2* *KD* rescued memory impairment in both male and female AD model flies (Figure 5B). This suggests that *Dgat2* modulation can influence cognitive function, with *KD* providing a protective effect in the context of AD, potentially through modulation of oligomer mediated synaptotoxicity. Interestingly, our sensory acuity test (Meschi et al. 2024) confirmed these trends, reinforcing the notion that *Dgat2* manipulation, particularly *KD*, can help preserve cognitive function in AD models. Regarding sleep, *Dgat2* *KD* increased total sleep and reduced sleep activity at 3 weeks of age (Figure 5C,D). These results indicate that *Dgat2* manipulation does not dramatically alter sleep behavior in younger flies, although the effects on sleep were age dependent (Kao et al. 2020). However, in older AD flies (7 weeks), sleep patterns were largely unchanged by *Dgat2* modulation, suggesting that the effects of lipid metabolism manipulation on sleep were less pronounced with age (Figure S9). These findings highlight the complexity of lipid metabolism's role in neurodegenerative diseases, with age and disease progression influencing the extent of *Dgat*2's impact on sleep, cognition, and motor function.

We further evaluated the effect of *Dgat2* *KD* on lipid metabolism and neuroinflammation in *Drosophila* and mous*e* models of AD. *Dgat2* *KD* significantly reduced lipid accumulation, a hallmark of AD pathology, and increased synapsin levels (Figure 6A–C). This increase in synapsin levels supports the idea that reducing lipid accumulation may help preserve synaptic integrity, a crucial aspect of cognitive function in AD models and potentially reduce oligomer‐mediated synaptic toxicity (Jain et al. 2021). We also observed reduced expression of *Upd1* and *Upd3* cytokines associated with neuroinflammation in AD (Figure S8). Interestingly, the expression of *Dome* and *Hop* receptors, which are integral to the JAK/STAT signaling pathway, remained unchanged, suggesting that *Dgat2* *KD's* effects on inflammation was mediated through lipid‐related mechanisms, not directly via the JAK/STAT pathway (Iglesias et al. 2017).

In conclusion, both panneuronal and glial‐specific expression of *App* ^*NLG*^ *and* Aβ42 led to severe impairments in locomotor and cognitive function, increased lipid accumulation, and decreased synapsin levels consistent with oligomer‐mediated synaptotoxicity. *Dgat2* *KD* partially rescued these detrimental effects, providing insight into the potential therapeutic relevance of *Dgat2* in AD through modulation of lipid oligomer interactions. Our findings suggest that targeting lipid metabolism, particularly through *Dgat2* modulation, may represent a therapeutic approach to reduce oligomeric Aβ toxicity in *Drosophila*. Further research into the molecular markers involved in these processes and the role of *Dgat2* modulation could provide valuable targets for mitigating neurodegeneration in the *App* ^*NLG‐F*^ mouse model (Figure 7).

## 4 Experimental Procedures

Due to space limitations, experimental procedures are described in the Supplementary section along with extended data in a separate PDF file.

## Author Contributions

Girish Melkani and Archana Yadav designed *Drosophila* related experiments including feedback from other authors. Jianhua Zhang, Josh Kramer, and Xiaosen Ouyang designed the mouse experiments. Archana Yadav performed most sleep and all geotaxis experiments. Archana Yadav performed the analysis of sleep parameters, qPCR experiments, and analysis of both *Drosophila* and mouse samples. Xiaosen Ouyang performed all immunofluorescence staining of mouse brain samples. Morgan Barkley performed most memory assays. John C. Watson processed all brain samples of the *Drosophila* brain and performed immunofluorescence staining and analyses with help from Girish Melkani. Kishore Madamanchi collected some sleep activity and memory data. Archana Yadav performed the analysis and analyzed all the data, including statistical analyses with help from Girish Melkani and Jianhua Zhang. Archana Yadav prepared the paper with Girish Melkani's input. All the authors reviewed the manuscript and provided their feedback.

## Funding

This work was partially supported by National Institutes of Health (NIH) grants AG065992 and RF1NS133378 to Girish C. Melkani, P30 AG050886 (Girish C. Melkani and Jianhua Zhang), T32 HL007457 (Josh Kramer), R01AG072895, R01ES034846, and R21AG081687 (Jianhua Zhang). This work is also supported by UAB Startup funds 3123226 and 3123227 to Girish C. Melkani.

## Conflicts of Interest

The authors declare no conflicts of interest.

## Supporting information


**Data S1:** acel70439‐sup‐0001‐Supinfo.pdf.

## Data Availability

The data that supports the findings of this study are available in the Supporting Information of this article.
