# Supplementary material for: Regulation of Lipid Dysmetabolism and Neuroinflammation Progression Linked With Alzheimer's Disease Through Modulation of Dgat2
Source: Aging Cell. 2026 Mar 15;25(3):e70439. doi: 10.1111/acel.70439 (PMC13093376; doi:10.1111/acel.70439)
Supplement: Supplementary file 1 — Data S1: acel70439‐sup‐0001‐Supinfo.pdf. [file ACEL-25-e70439-s001.pdf]

Regulation of Lipid Dysmetabolism and Neuroinflammation Progression Linked with Alzheimer's Disease Through Modulation of Dgat2

Archana Yadav[1], Xiaosen Ouyang[1], Morgan Barkley[1], John C Watson[1], Kishore Madamanchi[1], Josh Kramer[1], Jianhua Zhang[1, 2], Girish C. Melkani[1, 2]

[1]Department of Pathology, Division of Molecular and Cellular Pathology, Heersink School of Medicine, University of Alabama at Birmingham, Birmingham, AL 35294, USA. [2] UAB Nathan Shock Center, Birmingham, AL 35294

[2]Corresponding Address: Department of Pathology, Division of Molecular and Cellular Pathology, School of Medicine, University of Alabama at Birmingham, AL 35294, USA. Tel.: 1-205-996-0591; Fax: 1-205-934-7447; E-mail: girishmelkani@uabmc.edu (GCM)

Supplementary Data: A Single file contains materials and methods along with references, Supplementary Figures S1-10 and figure legends.

1. Materials and Methods

1.1. Drosophila models

Flies were maintained on a standard regular diet consisting of agar 11 g/L, active dry yeast 30 g/L, yellow cornmeal 55 g/L, molasses 72 mL/L, 10% nipagen 8 mL/L, and propionic acid 6 mL/L. Flies were housed at 23 °C, and 50% humidity under a 12-h light/12-h dark (LD) cycle and changed to new food every 3-4 days (Livelo et al., 2023; Villanueva et al., 2019). We used the following driver lines from Bloomington Drosophila Stock Center (BDSC), *Elav*-Gal4 (BL#458) for panneuronal expression (Koushika et al., 1996), *GLaz*-Gal4 (BL#8765) for glial expression (Sanchez et al., 2006), and OK107-Gal4 (BL#854) for mushroom bodies (Aso et al., 2009). We used the following UAS lines: UAS-App^{NLG} (human APP carrying the familial Alzheimer's Swedish K670N, M671L, and Arctic E693G mutations, BL#33794) and UAS-Aβ42 (BL#33769). As previously described, we have also used UAS-Dgat2 RNAi (BL#107788), and UAS-GFP (BL#5431) (Guo et al., 2024; Livelo et al., 2025; Moraes et al., 2024). As recently reported, we used the UAS-Gal4 system to drive tissue-specific expression or *knockdown (KD)* of *Dgat2* in specific brain regions (Moraes et al., 2024). Flies with *Elav> DGAT2* or *Elav > Dgat2 RNAi* (with a balancer) were crossed with UAS-App^{NLG}, UAS-Aβ42, or control constructs to investigate their effects. In addition to each UAS line driven under different drivers, the driver and UAS lines alone, and GFP overexpression serve as controls, as previously reported (Guo et al., 2024; Livelo et al., 2025; Moraes et al., 2024). Progeny from the above crosses were collected at eclosion, separated by sex, maintained at a density of 25 flies/vial on the diet mentioned above. As indicated in the results section, experiments were performed on 3-week-old (early middle age) and 7-week-old (old age) male or female flies as previously reported (Guo et al., 2024; Livelo et al., 2025; Moraes et al., 2024).

1.2. Mice

All mouse breeding procedures and experiments were approved by the Institutional Animal Care and Use Committee (IACUC) at the University of Alabama at Birmingham. Mice were maintained in the Laboratory Animal Shared Resource under controlled air, light, and temperature conditions with ad libitum access to food and water. The $App^{NLG\text{-}F}$

mice carry three familial AD-associated mutations in the APP gene: Swedish mutation *(NL),* which enhances total Aβ production; Iberian mutation *(F),* which increases the Aβ42/ Aβ40 ratio; and Arctic mutation *(G),* which promotes Aβ aggregation by facilitating oligomerization and reducing proteolytic degradation (Yokoyama et al., 2022). $App^{NLG\text{-}F}$ and WT (controls) mice, maintained in a C57BL/6J background, were euthanized by cervical dislocation at the ages of 4 months (n=3) and 15 months (n=3). Half of the brain was used for immunohistochemistry, and half of the cortex was used for RNA analyses.

1.3. Drosophila olfactory aversion training to test memory

Flies were trained using the olfactory aversive conditioning method as previously described (Chocron et al., 2022; Munkacsy et al., 2019). Approximately 30-40 flies per group were used for each genotype. Each genotype was exposed to two neutral odors (3-octanol and 4-methylcyclohexanol) prepared as a 1/10 dilution in mineral oil. A current of 100-V and 90-Hz was used as a shock reinforcer. Memory conditioning was performed using a T-maze apparatus (CelExplorer Labs). The olfactory aversive conditioning consists of three phases: naïve, training, and testing. During the naïve phase, flies are exposed to both odors for 3 minutes and 30 seconds to determine their odor preference based on the odor presented in each odor chamber. A single training round consists of exposing flies to odors paired with electric shock for 2 minutes, followed by 2 minutes of exposure to odors paired without a shock. Depending on the flies naïve odor preference, electrical shocking will be paired with their preferred odor. After three training rounds, flies are given 10 minutes to recover. During the testing phase, flies are given 3 minutes and 30 seconds to choose between odor chambers. Once time has expired, T maze chambers are sealed, and the number of flies in each chamber is scored. A performance index (PI) was calculated based on flies' avoidance of odor paired with electrical shock. To confirm these findings, we conducted a sensory acuity test based on avoidance index calculations, as previously described (Meschi et al., 2024).

1.4. Drosophila locomotion assay

To evaluate the panneuronal and glial-specific expression of APP mutation and the impact of *Dgat2 KD* on locomotor capability, we have developed a new 3D-printed device to analyze locomotive performance, using machine learning based analysis methods

(Melkani et al., 2026). Briefly, the device holds 12 vials and features a Raspberry Pi Camera aimed precisely at them, which is connected to a Raspberry Pi 4B and a monitor. A custom Python script developed in our lab is running on the Raspberry Pi, controlling both the camera and the attached motor system. The video was recorded after tapping the device, and the Faster RCNN deep learning model was used to detect vial boundaries in the video. The flies' climbing behavior was recorded on video for later analysis. Followed by Python analysis processes video frames for fly detection and detection across time for average climbing were plotted.

1.5. Drosophila sleep circadian activity

Sleep-wake and circadian activity behavior were recorded using the Drosophila Activity Monitor (DAM, TriKinetics Inc MA, USA) system in a 12 h Light: 12 h Dark (LD) cycle at 25°C (Abou Daya et al., 2025). We used 3-week and 7-week-old male and female progeny of *Elav*-Gal4 and *GLaz*-Gal4 with control lines of each of the four genes *(*w^{1118}*,* GFP, App^{NLG}*,* Aβ42). Drosophila activity (or wake) is measured by infrared beam crosses in the DAM system. Data was analyzed using Clock Lab and R Studio. Custom R scripts and methodology used with R Studio can be found at https://github.com/jameswalkerlab/Gill_et.al. Non-parametric One-way ANOVA with multiple comparisons, Kruskal-Wallis test for DAM system data was performed using GraphPad Prism. *Drosophila* sleep was defined by a period of at least 5 minutes of inactivity, demonstrated by zero beam breaks recorded. Average sleep per 24 hours (Zeitgeber Time, ZT) is a standardized way of measuring time within a circadian cycle, where ZT0 represents the beginning of the light phase and ZT12 marks the start of the dark phase), of each genotype was calculated. Five days were used for analysis of 3-week-old flies, and 3 days were used for 7-week-old flies due to decreased viability in older flies. Sleep bouts were quantified by counting the number of periods of sleep as defined above. Sleep bout length was quantified by measuring the length of each sleep bout. Data for daytime sleep is from ZT0 to ZT12, and nighttime sleep is from ZT12 to ZT24.

1.6. Histological analysis of Drosophila brain samples

The impact of APP mutation and Aβ42, with and without *Dgat2* modulation, on lipid and synapsin alterations was investigated as previously described (Watson et al., 2025). Briefly, fly heads were excised under a dissecting microscope, then fixed in 4% paraformaldehyde (PFA) in phosphate-buffered saline (PBS) for 15 minutes at room temperature with gentle agitation. The heads were washed three times for 10 minutes in 1× PBS. After the final wash, heads were transferred to a 10% sucrose in PBS solution, ensuring full saturation overnight. They were next arranged in a mold using an optimal temperature (OCT) compound. Once frozen in place, the samples were sectioned at 20μm using a Leica CM3050 S cryostat and transferred onto warmed glass slides (Fisher #15-188-48). After a 30-minute drying period and application of a hydrophobic barrier, slides were washed three times for 5 minutes with 1× PBS to remove the dried OCT. The slides were then blocked with 3% BSA in TBS solution for 30 minutes and incubated overnight at 4°C or for 1 hour at room temperature with primary synapsin antibody (1:250, UI Developmental Studies Hybridoma Bank #3C11) in 3% BSA in TBS. After incubation, slides were washed three times for 5 minutes with PBS and incubated for 1 hour at room temperature with an AlexaFluor-750 anti-mouse fluorescent secondary antibody (1:500, Thermo Fisher # A-21037) and lipid (1:100, Lipid Spot488, Biotinum #70065). Finally, slides were washed three times for 5 minutes with 1× PBS and mounted with Antifade Mounting Medium with DAPI (0.9μg/ ml, VECTASHIELD Vibrance H-1800). After overnight setting, multichannel fluorescence images were captured using an Olympus BX63 fluorescence microscope and analyzed with CellSens software at 10× magnification to view one section of the head per image.

The DAPI channel was used to define regions of interest (ROIs), as it clearly distinguishes between anatomical sub-regions in the head (Watson et al., 2025). Multiple sections were imaged for each fly, and the average fluorescent intensity as well as average object count, and area were compiled across sections to determine the overall lipid accumulation value per group. The 488 (lipid) channel was thresholded to reduce the background signal and the minimum object size for detection was set to 20 pixels. The 750 (synapsin) channels remained thresholded to retain integrity during intensity comparison between individual subjects as well as conditions. All thresholding values and background filters were applied in batches across all images before data collection.

Selection of multiple sections (n≥3) per fly and multiple flies within each condition (n≥3) ensured all areas of the head and brain are equally weighted during comparison.

2.7. Mice brain staining, and image quantification

One brain hemisphere was placed in 4% paraformaldehyde overnight at 4ºC, followed by sucrose saturation. The brain sample was frozen in OCT blocks and stored at -80ºC. 10 µm tissue was cryosectioned. Nile red (Sigma: N3013) and LipidSpot™ 488 (Biotium NC1669425) were used for Lipid Droplet Staining following the company's instructions. GFAP (Dako: Z0334, 1:20,000) or IBA11/AIF-1 (Cell Signaling Technology 17198S: 1:100) were paired with secondary Alexa Fluor 488 (Invitrogen cat# A11008, 1:1,000). DGAT2 (Protein Tech: 17100-1-AP, 1:50), and ADRP/Perilipin 2 (Protein Tech: 15294-1-AP, 1:500). Hoechst (Fisher PI62249) was used as a nuclear stain. Images were acquired using the Keyence BZ-X810 microscope, and ImageJ was used for image analysis.

2.8. Real-time quantitative PCR in Drosophila and mouse brain samples

As previously reported (Moraes et al., 2024), heads from 3-week-old and 7-week-old flies were collected and rapidly frozen. RNA extraction was performed using the Zymo Research Quick-RNA Microprep Kit (catalog R1051, Zymo Research, Irvine, CA, USA), which included on-column DNase I digestion. Quantitative PCR was conducted using the Sso Advanced Universal SYBR Green Supermix from Bio-Rad, employing the BIO-RAD CFX Opus Real-Time PCR System. Expression levels were standardized using the 60s ribosomal protein (Rpl11) as a reference; three biological replicates were used with 8-10 flies each. Primers for qPCR are listed below:

Upd1-F: CAGCGCACGTGAAATAGCAT; Upd1-R: CGAGTCCTGAGGTAAGGGGA;
Upd2-F: AGCGTCGTGATGCCATTCA; Upd2-R: GCGATACGGATTGACATCGAA;
Upd3-F: ATCCCCTGAAGCACCTACAGA; Upd3-R: CAGTCCAGATGCGTACTGCTG;
Dome-F: CTCACGTCTCGACTGGGAAC; Dome-R: AGAATGGTGCTTGTCAGGCA;
Hop-F: CACCACCAACACCAATTC; Hop-R: GGAACGTCGTTTGGCCTTCT;
Stat92e-F: CCTCGGTATGGTCACACCC; Stat92e-R: TGCCAAACTCATTGAGGGACT;
Eiger-F: GATGGTCTGGATTCCATTGC; Eiger-R: TAGTCTGCGCCAACATCATC;
Imd-F: TCAGCGACCCAAACTACAATTC; Imd-R: TTGTCTGGACGTTACTGAGAGT;
Reaper-F: TGGCATTCTACATACCCGATCA;
Reaper-R: CCAGGAATCTCCACTGTGACT;

Hid-F: CACCGACCAAGTGCTATACG; Hid-R: GGCGGATACTGGAAGATTTGC;
Desat2-F: GTCGGCTACCCCTAGTCTGG; Desat2-F: TCGCCCTTGTGAATATGGAGT;
Srebp-F: ACCAACAGCCACCATACATCA; Srebp-R: AGACAAAGCTACTGCCCAGAG;
Dgat2-F: ATCCGTTGTGGATGGCAATG;
Dgat2-R: GGGAAAGTAATCACGATAGTGGC;
Rpl11-F: CGATCTGGGCATCAAGTACGA; Rpl11-R: TTGCGCTTCCTGTGGTTCAC;
Results are presented as $2^{-\Delta\Delta Ct}$ values normalized to the expression of Rpl11 and. All reactions were performed in triplicate.

Mouse RNA extraction was performed by using RNeasy Plus Mini Kit (Qiagen, Cat#: 74134) from isolated mouse cortex samples. cDNA synthesis was performed using a High-Capacity cDNA Reverse Transcription Kit (Thermo Fisher Scientific, Cat#: 4368814). Primers for mouse qPCR are listed below:
Bin1-F: TTCGGACCTATCTGGCTTCTG; Bin1-R: CCTCCTGAAGACACTCACTCA;
Abca7-F: AATTACACCTATCGACGGAGACA; Abca7-R: TGACGGACAGCCACTAGGA;
Epha1-F: AGGAAGTCACTCTAATGGACACA;
Epha1-R: CCTCACTCCACCCAGTCTCT;
Rhbdf2-F: GCCCACACCGTATCTGTTCTG;
Rhbdf2-R: GATGCCAGTTTTGTCGCTTGC;
Apoe-F: GACCCAGCAAATACGCCTG; Apoe-R: CATGTCTTCCACTATTGGCTCG;
Srebf1-F: TGACCCGGCTATTCCGTGA; Srebf1-R: CTGGGCTGAGCAATACAGTTC;
Eda-F: AGTGCTCAATGACTGGTCTCG; Eda-R: CGCTGCGGGGATGTAGTTTA;
Stat5b-F: CGATGCCCTTCACCAGATG; Stat5b-R: AGCTGGGTGGCCTTAATGTTC;
Ptprq-F: ATTTCTGCCACAACCTACAGC;
Ptprq-R: GGAGGGGTATTCCATGAAAGGAG;
Jak2-F: TTGTGGTATTACGCCTGTGTATC; Jak2-R: ATGCCTGGTTGACTCGTCTAT;
Ripk1-F: AGAAGAAGGGAACTATTCGCTGG;
Ripk1-R: CATCTATCTGGGTCTTTAGCACG;
Scd1-F: TTCTTGCGATACACTCTGGTGC; Scd1-R: CGGGATTGAATGTTCTTGTCGT;
Dgat2-F: TTCCTGGCATAAGGCCCTATT; Dgat2-R: CCTCCAGACATCAGGTACTCG;
Results are presented as $2^{-\Delta\Delta Ct}$ values normalized to the expression of β-actin.

2.9. Statistical analysis

Significance of climbing ability was determined using two-way ANOVA with multiple comparisons done with Uncorrected Fisher's LSD test. Significance was based on a chi-square test for olfactory aversion training, and the difference between fly decisions, and flies avoiding shock odor were analyzed with one-way ANOVA with Tukey's multiple comparisons test. Lipid droplet size and density differences, and synaptic loss were performed by one-way ANOVA with Dunnett's multiple comparisons test for *Drosophila.* Relative mean intensity of lipid, Gfap and Iba1 was measured using two-way ANOVA with Tukey's multiple comparisons test for young and old mouse samples. For sleep activity analysis, differences between samples were determined using non-parametric one-way ANOVA with multiple comparisons done with the Kruskal-Walli's test. Quantitative PCR analyses were made using one-way ANOVA with Tukey's multiple comparisons test performed for *Drosophila* samples, and two-way ANOVA with multiple comparisons done with Uncorrected Fisher's LSD test was performed for young/ old mouse samples. Bar graphs show mean ± SD. All statistical analyses were performed with GraphPad Prism 10. Differences were significant at values of *$p < 0.05$; **$p < 0.01$; *** $p < 0.001$, and **** $p < 0.0001$.

References

Abou Daya, F., Mandigo, T., Ober, L., Patel, D., Maher, M., Math, S., Tchio, C., Walker, J. A., Saxena, R., & Melkani, G. C. (2025). Identifying links between cardiovascular disease and insomnia by modeling genes from a pleiotropic locus. *Dis Model Mech*, *18*(5). https://doi.org/10.1242/dmm.052139

Aso, Y., Grubel, K., Busch, S., Friedrich, A. B., Siwanowicz, I., & Tanimoto, H. (2009). The mushroom body of adult Drosophila characterized by GAL4 drivers. *J Neurogenet*, *23*(1-2), 156–172. https://doi.org/10.1080/01677060802471718

Chocron, E. S., Munkacsy, E., Kim, H. S., Karpowicz, P., Jiang, N., Van Skike, C. E., DeRosa, N., Banh, A. Q., Palavicini, J. P., Wityk, P., Kalinowski, L., Galvan, V., Osmulski, P. A., Jankowska, E., Gaczynska, M., & Pickering, A. M. (2022). Genetic and pharmacologic proteasome augmentation ameliorates Alzheimer's-like pathology in mouse and fly APP overexpression models. *Sci Adv*, *8*(23), eabk2252. https://doi.org/10.1126/sciadv.abk2252

Guo, Y., Abou Daya, F., Le, H. D., Panda, S., & Melkani, G. C. (2024). Diurnal expression of Dgat2 induced by time-restricted feeding maintains cardiac health in the Drosophila model of circadian disruption. *Aging Cell*, *23*(7), e14169. https://doi.org/10.1111/acel.14169

Koushika, S. P., Lisbin, M. J., & White, K. (1996). ELAV, a Drosophila neuron-specific protein, mediates the generation of an alternatively spliced neural protein isoform. *Curr Biol*, *6*(12), 1634–1641. https://doi.org/10.1016/s0960-9822(02)70787-2

Livelo, C., Guo, Y., Abou Daya, F., Rajasekaran, V., Varshney, S., Le, H. D., Barnes, S., Panda, S., & Melkani, G. C. (2023). Time-restricted feeding promotes muscle function through

purine cycle and AMPK signaling in Drosophila obesity models. *Nat Commun*, *14*(1), 949. https://doi.org/10.1038/s41467-023-36474-4

Livelo, C., Guo, Y., Madhanagopal, J., Morrow, C., & Melkani, G. C. (2025). Time-restricted feeding mediated modulation of microbiota leads to changes in muscle physiology in Drosophila obesity models. *Aging Cell*, *24*(2), e14382. https://doi.org/10.1111/acel.14382

Melkani, D., Harnwal, N., Desai, S., Patel, D., & Melkani, G. (2026). Design and Implementation of an Automated Drosophila Locomotor Assay Using Computer Vision Tracking. *Res Sq*. https://doi.org/10.21203/rs.3.rs-8769384/v1

Meschi, E., Duquenoy, L., Otto, N., Dempsey, G., & Waddell, S. (2024). Compensatory enhancement of input maintains aversive dopaminergic reinforcement in hungry Drosophila. *Neuron*, *112*(14), 2315–2332 e2318. https://doi.org/10.1016/j.neuron.2024.04.035

Moraes, R. C. M., Roth, J. R., Mao, H., Crawley, S. R., Xu, B. P., Watson, J. C., & Melkani, G. C. (2024). Apolipoprotein E Induces Lipid Accumulation Through Dgat2 That Is Prevented with Time-Restricted Feeding in Drosophila. *Genes (Basel)*, *15*(11). https://doi.org/10.3390/genes15111376

Munkacsy, E., Chocron, E. S., Quintanilla, L., Gendron, C. M., Pletcher, S. D., & Pickering, A. M. (2019). Neuronal-specific proteasome augmentation via Prosbeta5 overexpression extends lifespan and reduces age-related cognitive decline. *Aging Cell*, *18*(5), e13005. https://doi.org/10.1111/acel.13005

Sanchez, D., Lopez-Arias, B., Torroja, L., Canal, I., Wang, X., Bastiani, M. J., & Ganfornina, M. D. (2006). Loss of glial lazarillo, a homolog of apolipoprotein D, reduces lifespan and stress resistance in Drosophila. *Curr Biol*, *16*(7), 680–686. https://doi.org/10.1016/j.cub.2006.03.024

Villanueva, J. E., Livelo, C., Trujillo, A. S., Chandran, S., Woodworth, B., Andrade, L., Le, H. D., Manor, U., Panda, S., & Melkani, G. C. (2019). Time-restricted feeding restores muscle function in Drosophila models of obesity and circadian-rhythm disruption. *Nat Commun*, *10*(1), 2700. https://doi.org/10.1038/s41467-019-10563-9

Watson, J., Roth, J. R., & Melkani, G. C. (2025). Direct Cryosectioning of Drosophila Heads for Enhanced Brain Fluorescence Staining and Immunostaining. *J Vis Exp*(216). https://doi.org/10.3791/67791

Yokoyama, M., Kobayashi, H., Tatsumi, L., & Tomita, T. (2022). Mouse Models of Alzheimer's Disease. *Front Mol Neurosci*, *15*, 912995. https://doi.org/10.3389/fnmol.2022.912995

Supplemental Figure 1

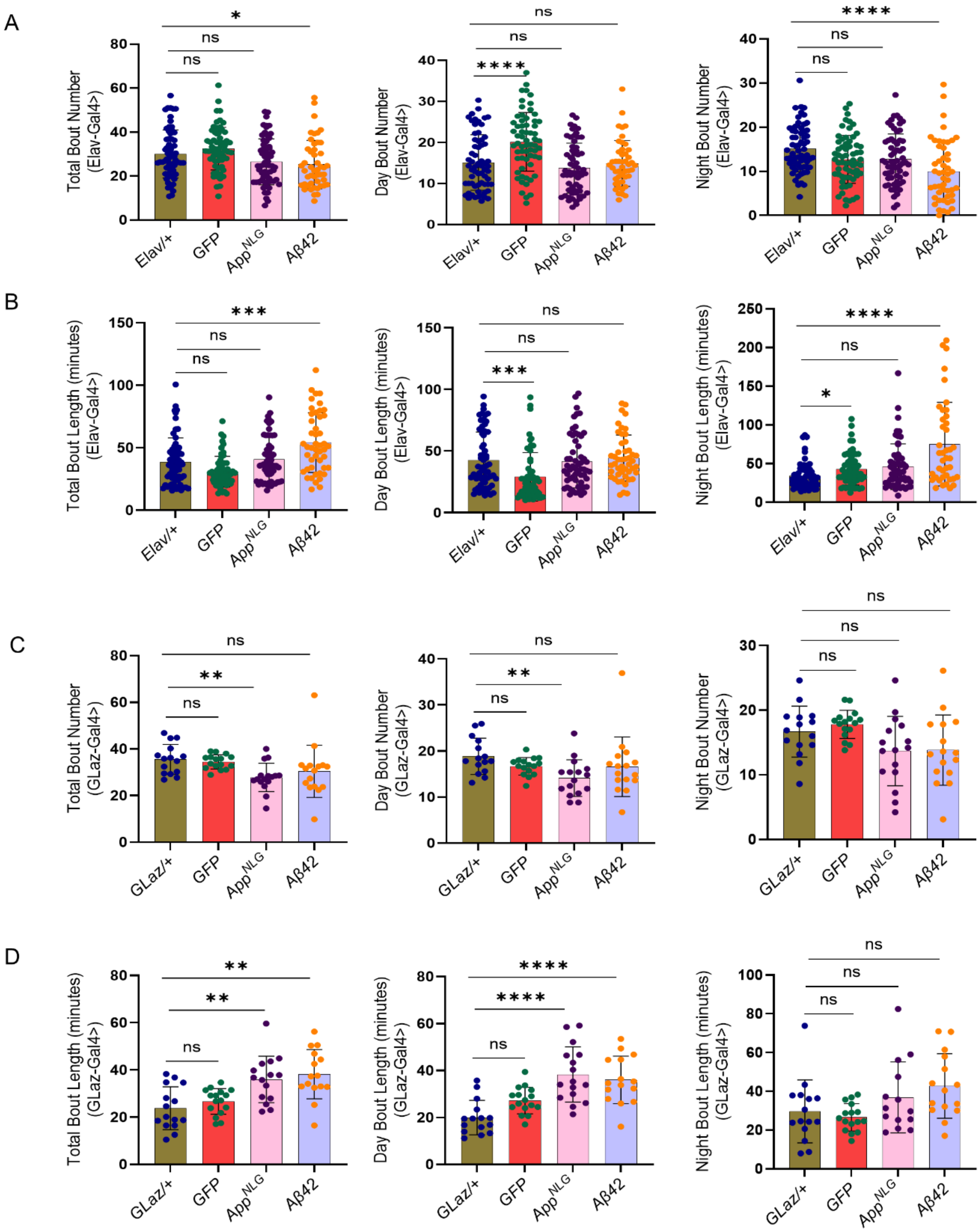

Supplemental Figure 1 | Panneuronal specific expression of Aβ42 led to compromised sleep activity, bout number, and bout length compared to *App*^{NLG} at 3-week. (A) Total, day and night sleep bout numbers in *Elav*-driven *App*^{NLG} and Aβ42 models. (B) Total, day and night sleep bout lengths in *Elav*-driven *App*^{NLG} and Aβ42 models only compared to their respective controls. (C) Total, day and night sleep bout numbers, (D) bout length in *GLaz*-driven *App*^{NLG} and Aβ42 models. All experiments were performed in 3-week-old males only. Data=mean ± SD. Non-parametric One-way ANOVA with multiple comparisons, done with the Kruskal-Wallis test, was performed. Each dot represents the number of flies. *p<0.05, **p<0.01, ***p<0.001, and ****p<0.0001; ns, not significant (an asterisk denotes significance for the average of all three replicates). Raw data and p values are provided in the source data.

Supplemental Figure 2

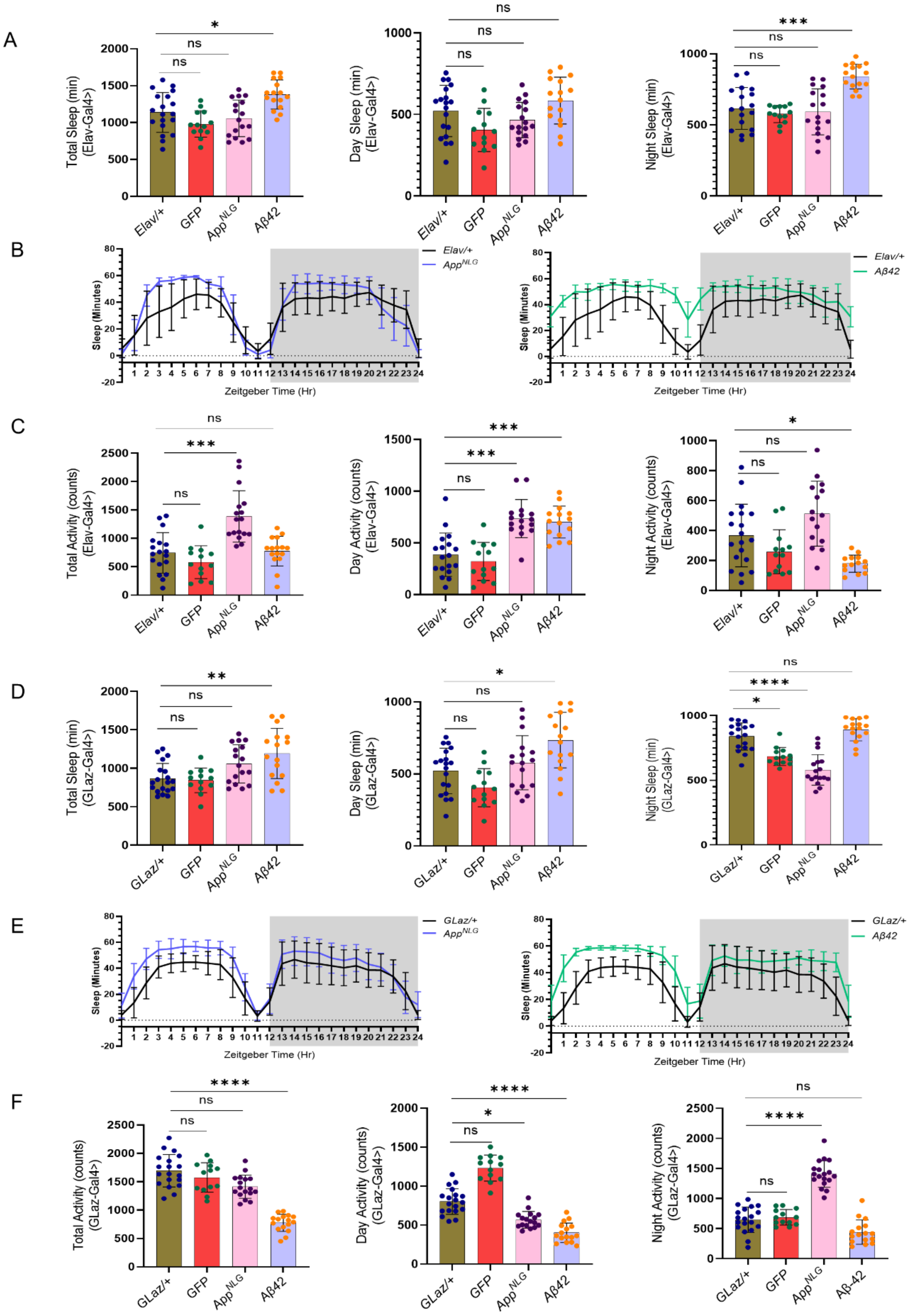

Supplemental Figure 2 | Panneuronal and glial-specific expression of Aβ42 led to compromised seep quality compared at 7 weeks. (A) Total, day and night sleep (in minutes) in *Elav*-driven *App*^{NLG} and Aβ42 models. (B) Sleep profiles at different zeitgeber time (hr.) in Elav-driven *App*^{NLG} and Aβ42 models only compared to their respective controls. (C) Total, day and night sleep activity (counts) in *Elav*-driven *App*^{NLG} and Aβ42 models. (D) Total, day and night sleep in (in minutes) in *GLaz*-driven *App*^{NLG} and Aβ42 models**.** (E) Sleep profiles at different zeitgeber time (hr.) in *GLaz*-driven *App*^{NLG} and Aβ42 models only compared to their respective controls. (F) Total, day and night sleep activity (counts) in *GLaz*-driven *App*^{NLG} and Aβ42 models. All experiments were performed in 7-week-old males only. Data=mean ± SD. Non-parametric One-way ANOVA with multiple comparisons done with Kruskal Wallis test was performed for sleep activity and sleep fragmentation. Each dot represents number of flies. *p<0.05, **p<0.01, ***p<0.001, and ****p<0.0001; ns, not significant (an asterisk denotes significance for the average of all three replicates). Raw data and p values are provided in the source data.

Supplemental Figure 3

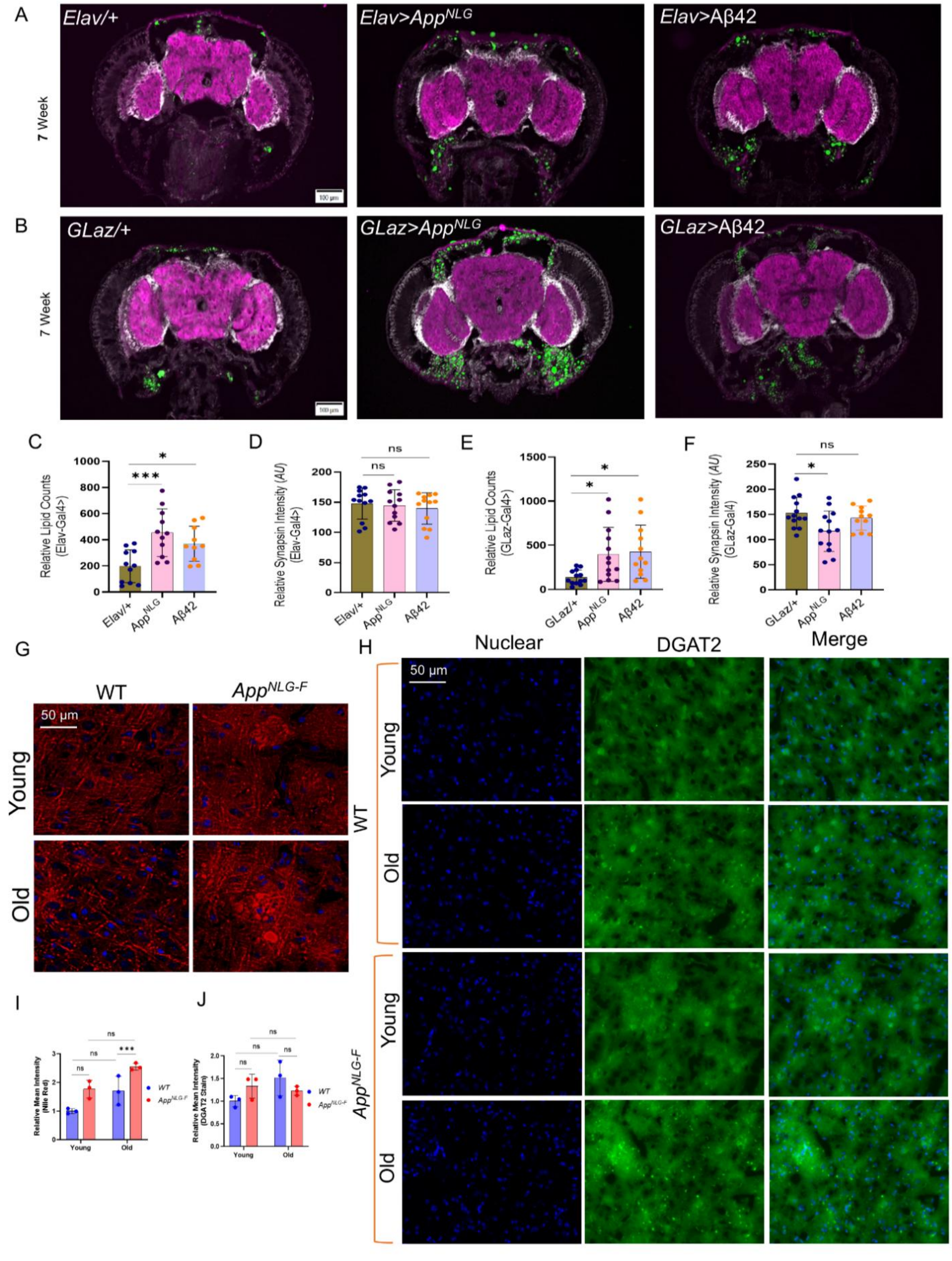

Supplemental Figure 3 | Age-dependent increase in lipid accumulation in *App^{NLG} and* Aβ42 *Drosophila* models, and *$App^{NLG\text{-}F}$* mice cortex, independent of *Dgat2* expression changes. (A, B) Representative images showing the expression of lipid accumulation (green, LipidSpot 488), synaptic loss (purple, anti SYNORF1), and DAPI (white), in the brains of *Elav* and *GLaz*-driven *App^{NLG}* and Aβ42 models flies. (C-F) Quantification of the expression level of lipid counts and synapsin intensity in *Elav* and *GLaz*-driven *App^{NLG}* and Aβ42 models. All experiments were performed in 7-week-old flies. (G) Representative image showing the intensity of Nile red (red), a marker of lipid accumulation, and DAPI (blue), in the cortical region of mouse brains from young and old wild-type (WT) and *$App^{NLG\text{-}F}$* models. (H) Representative image showing the level of DGAT2 immunostain, and DAPI (blue), in the mouse brains of young and old WT and *$App^{NLG\text{-}F}$* models. (I, J) Quantification of the intensity of Nile red (I) and relative mean intensity of DGAT2 (J) immunostain. Data=mean ± SD., with n=3 mice per group and 5-6 flies per group. Fold changes of fluorescence intensity were calculated relative to controls. One-way ANOVA with Tukey's multiple comparisons test was performed for the flies' data. Two-way ANOVA with multiple comparisons done with Uncorrected Fisher's LSD test, was performed for mouse data. *p<0.05, and ***p<0.00; ns, not significant (an asterisk denotes significance for the average of all three replicates). Raw data and p values are provided in the source data.

Supplemental Figure 4

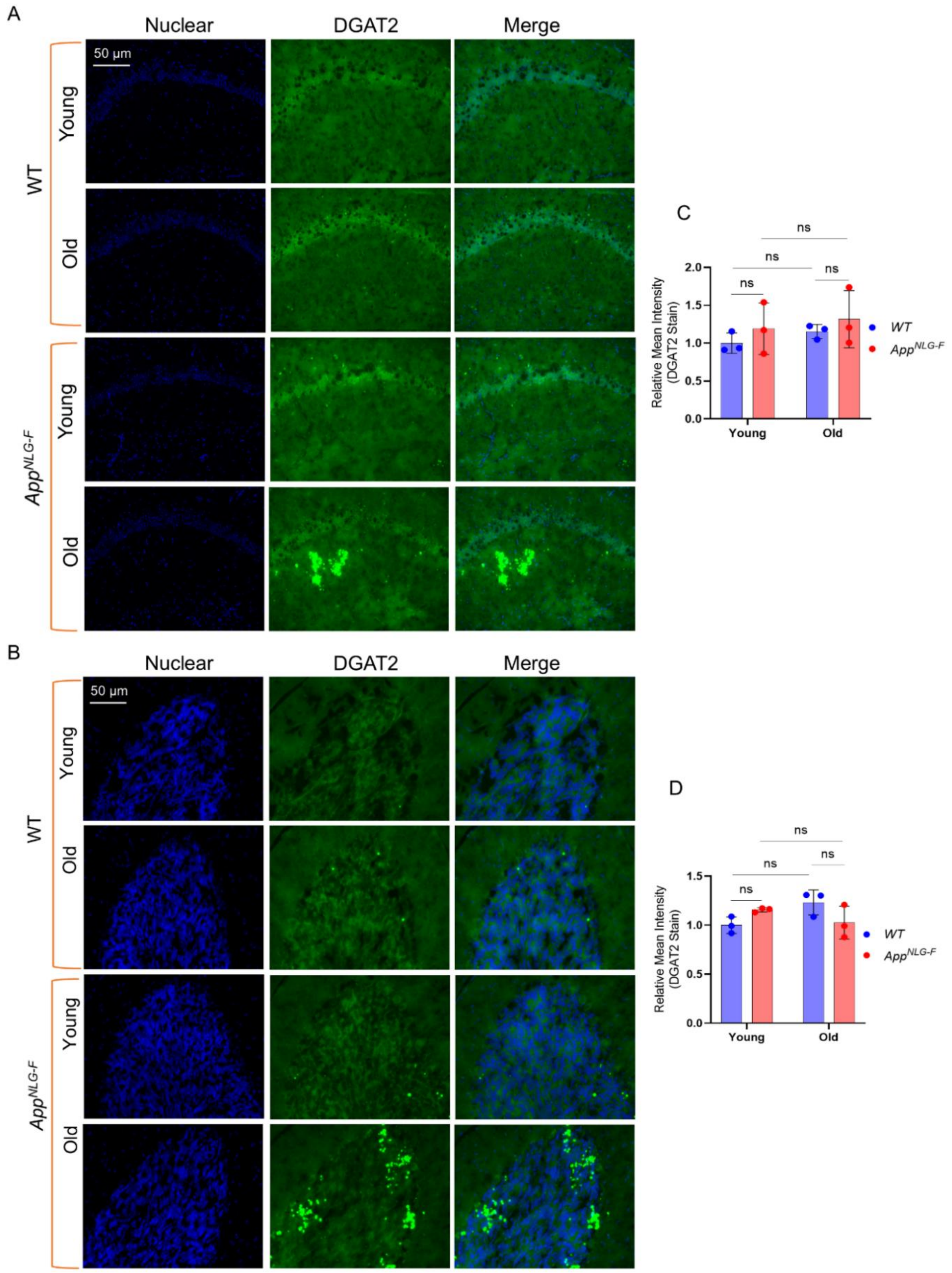

Supplemental Figure 4 | Unaltered DGAT2 stains in both hippocampus (CA1) and cerebellum (CB) of *App*$^{NLG\text{-}F}$ mice in old age. (A, B) Representative images showing the level of DGAT2 immunostain (green), and DAPI (blue), in the hippocampus (CA1, panel A, C) and cerebellum (CB, panel B, D) of mouse brains from young and old WT and *App*$^{NLG\text{-}F}$ models. (C, D) Quantification of the levels of DGAT2 immunostain in CA1 and CB**.** Data=mean ± SD., with n=3 mice per group. Fold changes of fluorescence intensity were calculated relative to controls. Two-way ANOVA with multiple comparisons done with Uncorrected Fisher's LSD test was performed for mouse data. No significant differences were observed among groups. ns, not significant (p>0.05). Raw data and p values are provided in the source data.

Supplemental Figure 5

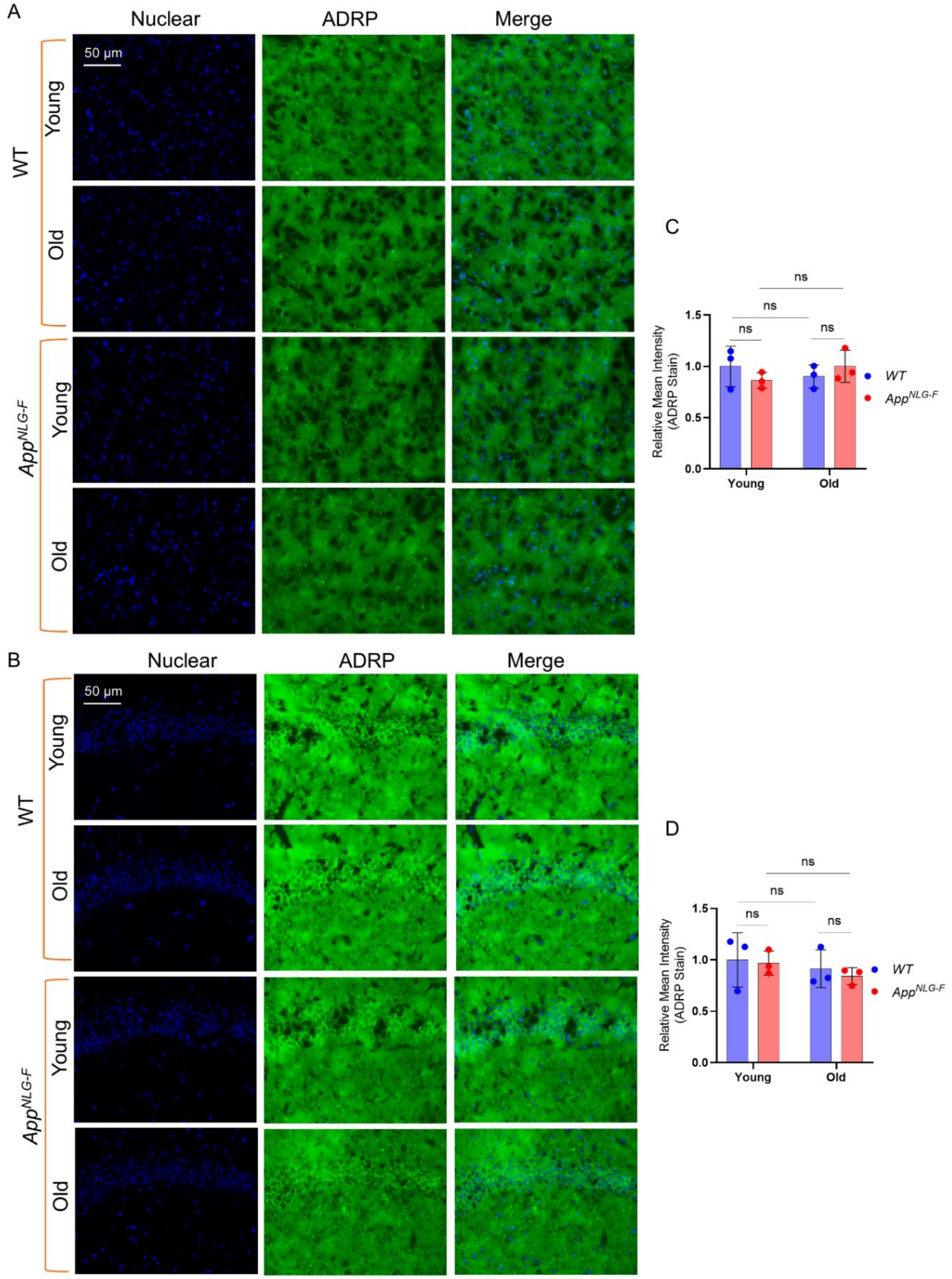

Supplemental Figure 5 | Unaltered adipose differentiation-related protein (ADRP) protein in both cortex and hippocampus of *App*$^{NLG\text{-}F}$ mice in old age. (A, B) Representative images showing the level of ADRP immunostain (green), and DAPI (blue), in the cortex (Ctx, panel A, C) and hippocampus (CA1, panel B, D) of mouse brains from young and old WT and *App*$^{NLG\text{-}F}$ mice. (C, D) quantification of the level of ADRP immunostain in cortex (C) and CA1 (D). Data=mean ± SD., with n=3 mice per group. Fold changes of fluorescence intensity were calculated relative to controls. Two-way ANOVA with multiple comparisons done with Uncorrected Fisher's LSD test was performed for mouse data. No significant differences were observed among groups. ns, not significant (p>0.05). Raw data and p values are provided in the source data.

Supplemental Figure 6

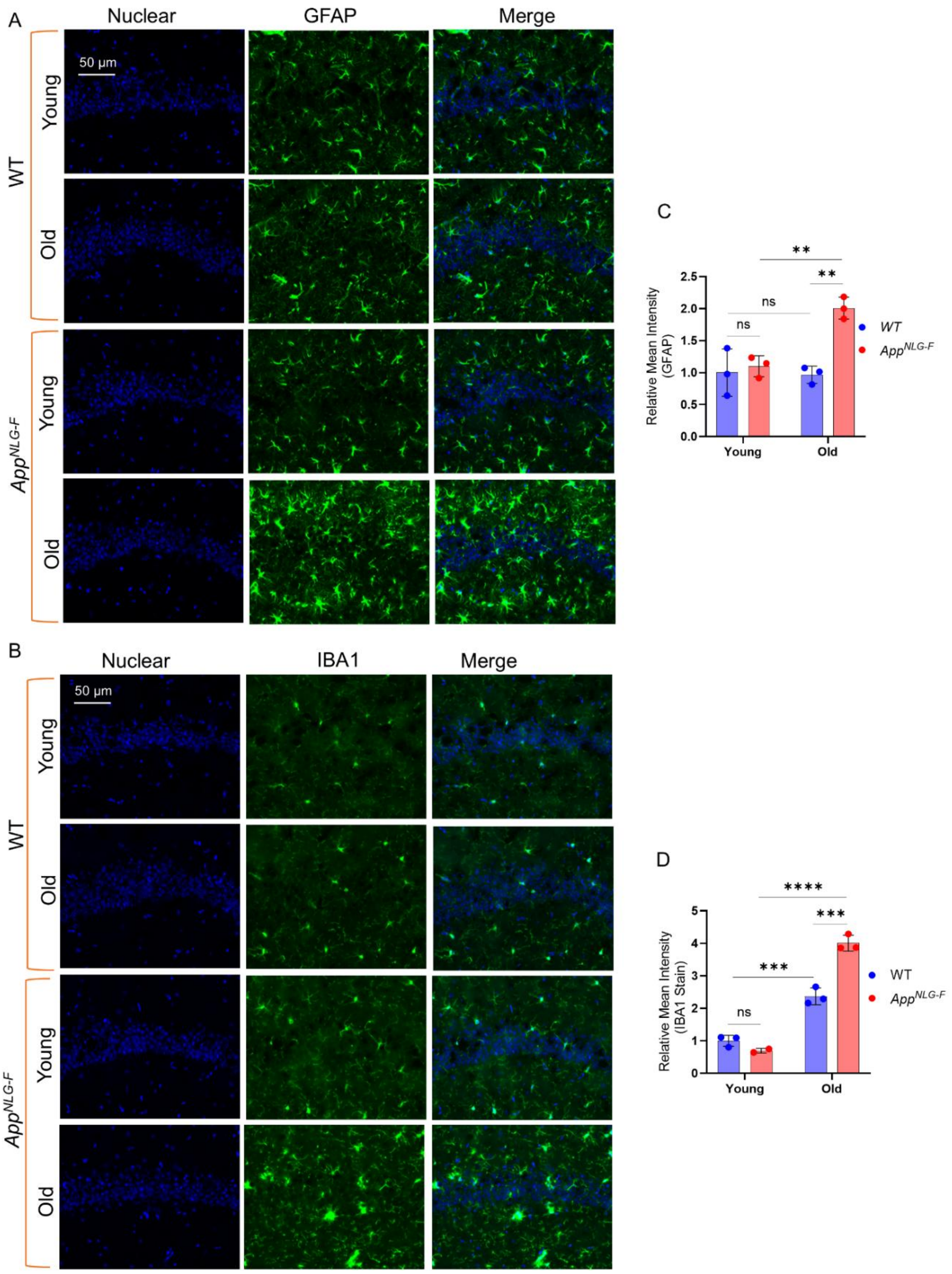

Supplemental Figure 6 | Age-dependent increase in astrocyte and microglial activation in *App*$^{NLG\text{-}F}$ mice, reflecting neuroinflammation. (A, B) Representative images showing the levels of GFAP and IBA1 immunostain (green), and DAPI (blue), in the hippocampus of young and old WT and *App*$^{NLG\text{-}F}$ mice. (c, d) Quantification of the level of GFAP (C) and IBA1 (D) immunostain in the cortex. Data=mean ± SD., with n=3 mice per group. Fold changes of fluorescence intensity were calculated relative to controls. Two-way ANOVA with multiple comparisons done with Uncorrected Fisher's LSD test was performed for mouse data. **p<0.01, ***p<0.001, and ****p<0.0001; ns, not significant (an asterisk denotes significance for the average of all three replicates). Raw data and p values are provided in the source data.

Supplemental Figure 7

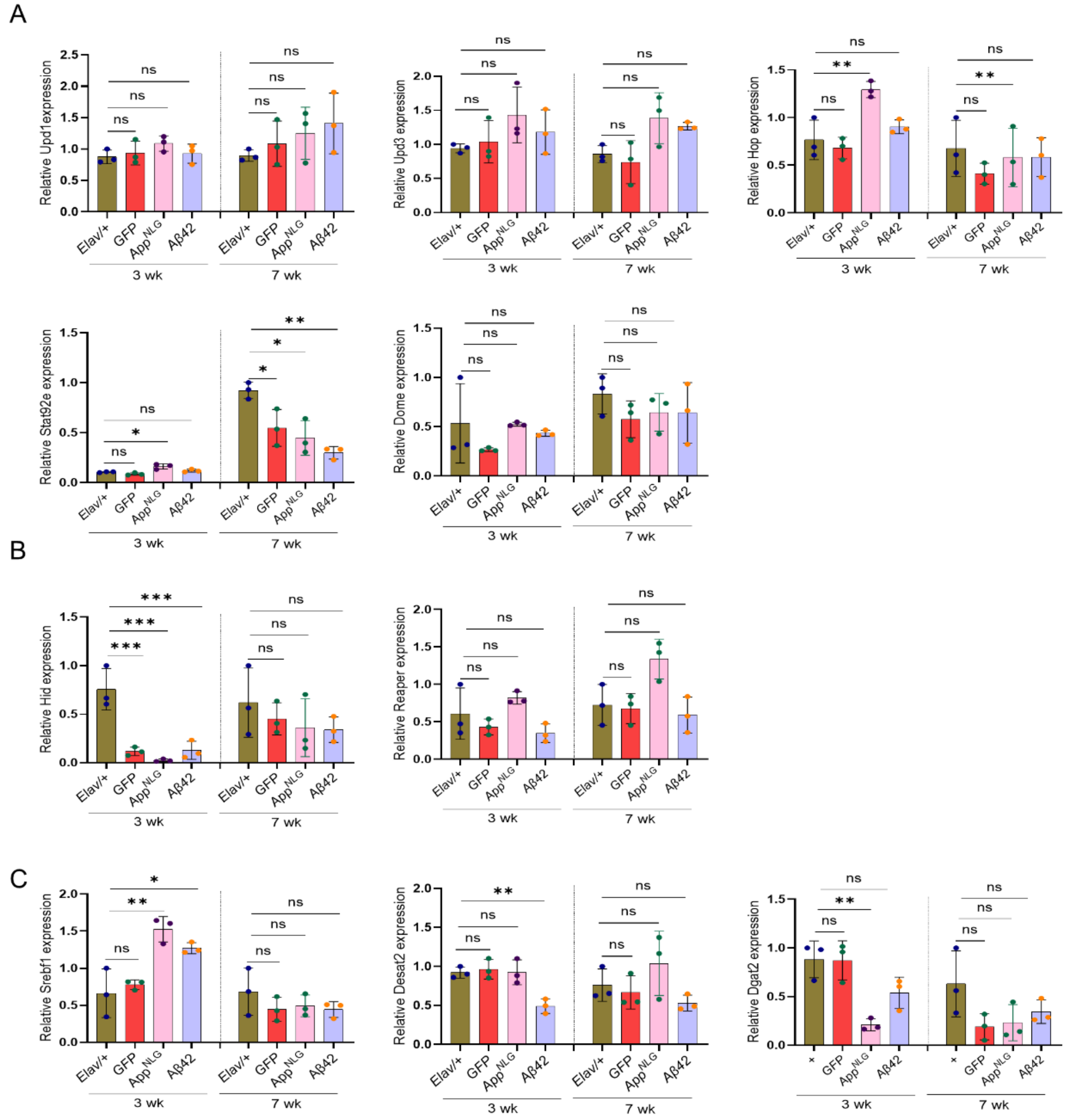

Supplemental Figure 7 | Chronic JAK/STAT signaling activation in *Drosophila App*^{NLG} and Aβ42 models leads to altered lipid metabolism. (A) The qPCR result of the relative expression level of Inflammatory genes, *Upd1, Upd2, Hop, Stat92e* and *Dome*. (B) Relative expression level of cell death markers *Hid* and *Reaper*. (C) Relative expression level of metabolic gene markers, *Srebf1*, *Desat2* and *Dgat2* in 3-week-old flies. Data=mean ± SD. One-way ANOVA with Tukey's multiple comparisons test was performed. *p<0.05, **p<0.01, and ***p<0.001; ns, not significant (an asterisk denotes significance for the average of all three replicates). Raw data and p values are provided in the source data.

Supplemental Figure 8

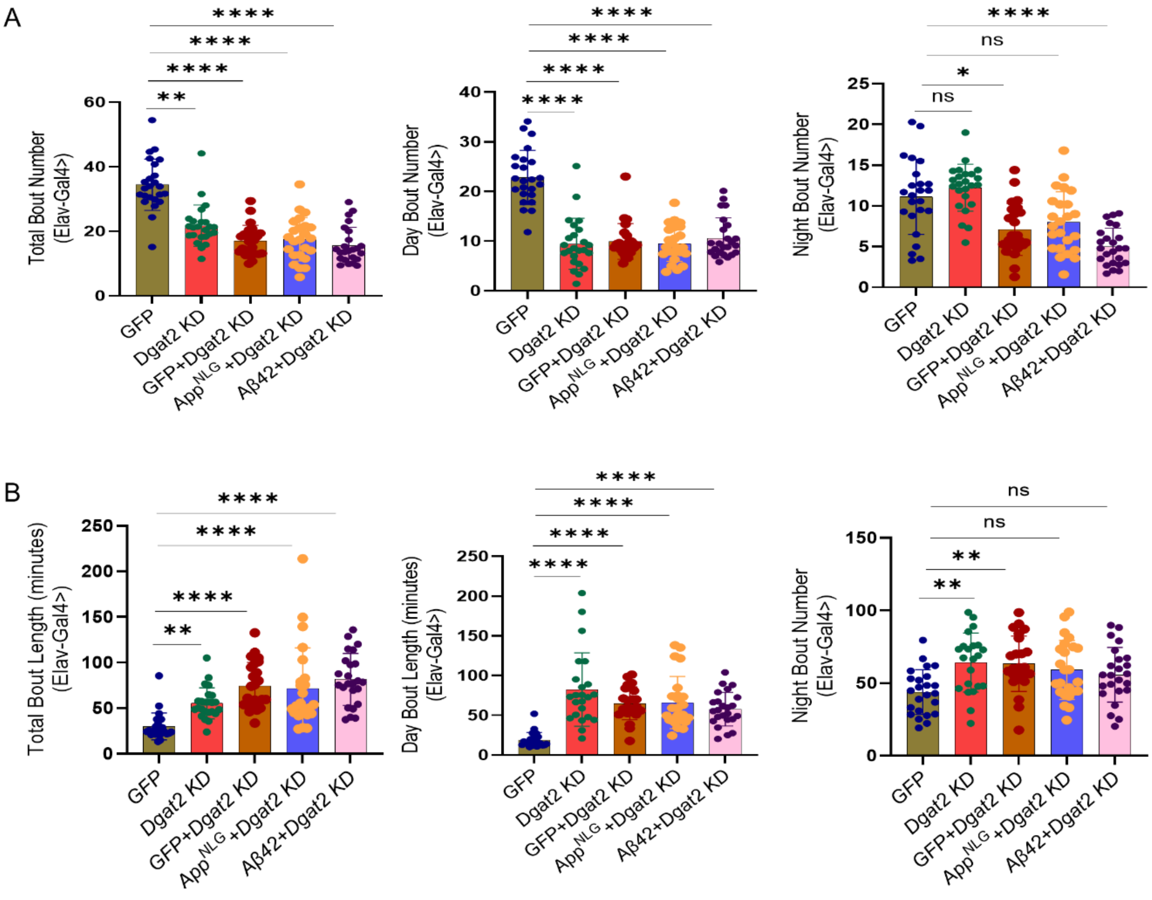

Supplemental Figure 8 | *Dgat2 knockdown* improves the sleep quality in *App*^{NLG} and Aβ42 models of flies at 3 weeks. (A) Total, day and night sleep bout numbers. (B) Total, day and night sleep bout length in *Elav*-driven *Dgat* KD in *App*^{NLG} and Aβ42 models**.** All experiments were done in 3-week-old males. Data=mean ± SD. Non-parametric One-way ANOVA with multiple comparisons, done with the Kruskal-Walli's test, was performed. Each dot represents the number of flies. **p*<0.05, ***p*<0.01, and *****p*<0.0001; ns, not significant (an asterisk denotes significance for the average of all three replicates). Raw data and *p* values are provided in the source data.

Supplemental Figure 9

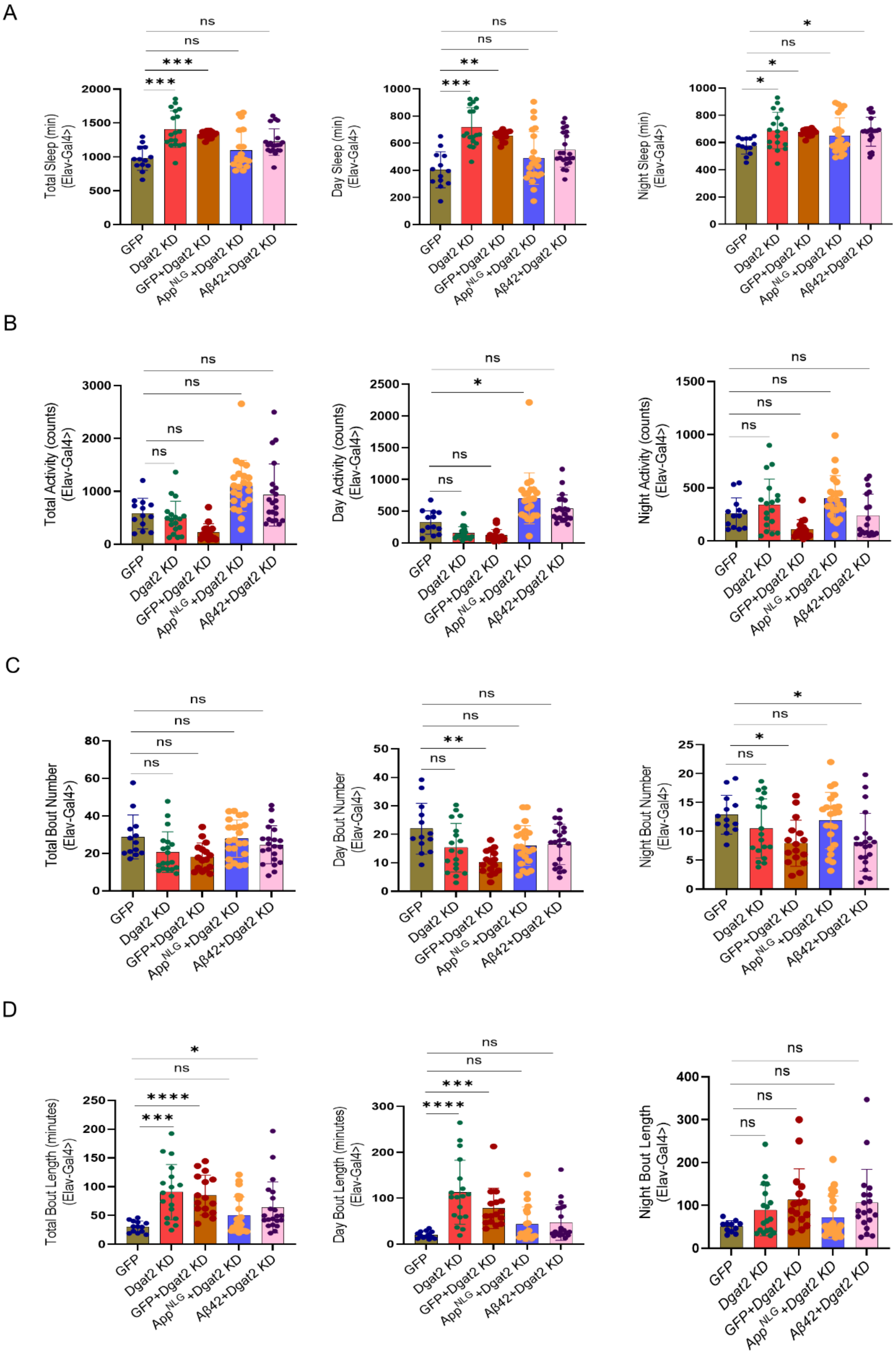

Supplemental Figure 9 |. ***Dgat2 knockdown* does not significantly affect sleep quality in AD models of flies at 7 weeks.** (A) Total, day and night sleep (in minutes). (B) Total, day, and night sleep activity (in counts). (C) Total, day and night sleep bout numbers. (D) Total, day and night sleep bout length in *Elav*-driven *Dgat KD* in App^{NLG} and Aβ42 models at 7-week-old males**.** Data=mean ± SD. Non-parametric One-way ANOVA with multiple comparisons done with Kruskal-Wallis test was performed for Sleep parameters. Each dot represents the number of flies. *p<0.05, **p<0.01, ***p<0.001 and ****p<0.0001; ns, not significant (an asterisk denotes significance for the average of all three replicates). Raw data and p values are provided in the source data.

Supplemental Figure 10

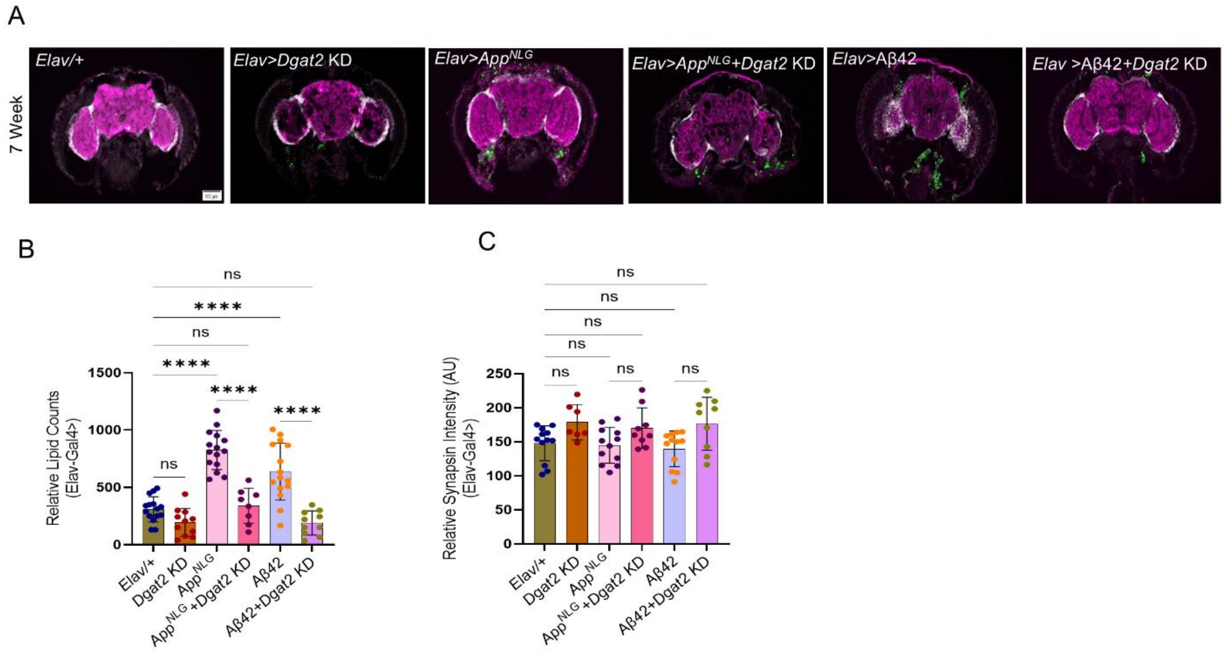

Supplemental Figure 10 | *Dgat2 knockdown* reduces lipid accumulation without affecting synapsin levels in *App^{NLG}* and Aβ42 *Drosophila* models. (A) Representative image showing the expression of lipid accumulation (green, LipidSpot488), synaptic loss (purple, anti-SYNORF1), a marker of neurodegeneration, and DAPI (white) in the brains of *Elav*-driven *Dgat2 KD* in *App^{NLG}* and Aβ42 models. (B, C) Quantification of the expression level of lipid counts and synapsin intensity in *Elav*-driven *Dgat2 KD* in *App^{NLG}* and Aβ42 models. All experiments were performed in 7-week-old flies. Data=mean ± SD., with n=5-6 flies per group. Fold changes of fluorescence intensity were calculated relative to controls. One-way ANOVA with Tukey's multiple comparisons test was performed for flies. ****p<0.0001, and ns, not significant (an asterisk denotes significance for the average of all three replicates). Raw data and p values are provided in the source data.
